# High proportion of genetic cases in patients with advanced cardiomyopathy including a novel homozygous *Plakophilin 2*-gene mutation

**DOI:** 10.1371/journal.pone.0189489

**Published:** 2017-12-18

**Authors:** Baerbel Klauke, Anna Gaertner-Rommel, Uwe Schulz, Astrid Kassner, Edzard zu Knyphausen, Thorsten Laser, Deniz Kececioglu, Lech Paluszkiewicz, Ute Blanz, Eugen Sandica, Antoon J. van den Bogaerdt, J. Peter van Tintelen, Jan Gummert, Hendrik Milting

**Affiliations:** 1 Erich and Hanna Klessmann Institute for Cardiovascular Research & Development (EHKI), Clinic for Thoracic and Cardiovascular Surgery, Heart and Diabetes Center NRW, Ruhr University Bochum, Bad Oeynhausen, Germany; 2 Clinic for Thoracic and Cardiovascular Surgery, Heart and Diabetes Center NRW, Ruhr University Bochum, Bad Oeynhausen, Germany; 3 Center for Congenital Heart Defects, Heart and Diabetes Center NRW, Ruhr University Bochum, Bad Oeynhausen, Germany; 4 Department of Surgery for Congenital Heart, Heart and Diabetes Center NRW, Ruhr University Bochum, Bad Oeynhausen, Germany; 5 Erasmus Medical Center, Department of Thoracic Surgery, Euro Heart Valve Bank, CA Rotterdam, The Netherlands; 6 Department of Clinical Genetics, University of Amsterdam, Academic Medical Center, AZ Amsterdam, The Netherlands; 7 Department of Genetics, University of Groningen, University Medical Center Groningen, GZ Groningen, The Netherlands; Indiana University, UNITED STATES

## Abstract

Cardiomyopathies might lead to end-stage heart disease with the requirement of drastic treatments like bridging up to transplant or heart transplantation. A not precisely known proportion of these diseases are genetically determined. We genotyped 43 index-patients (30 DCM, 10 ARVC, 3 RCM) with advanced or end stage cardiomyopathy using a gene panel which covered 46 known cardiomyopathy disease genes. Fifty-three variants with possible impact on disease in 33 patients were identified. Of these 27 (51%) were classified as likely pathogenic or pathogenic in the *MYH7*, *MYL2*, *MYL3*, *NEXN*, *TNNC1*, *TNNI3*, *DES*, *LMNA*, *PKP2*, *PLN*, *RBM20*, *TTN*, and *CRYAB* genes. Fifty-six percent (n = 24) of index-patients carried a likely pathogenic or pathogenic mutation. Of these 75% (n = 18) were familial and 25% (n = 6) sporadic cases. However, severe cardiomyopathy seemed to be not characterized by a specific mutation profile. Remarkably, we identified a novel homozygous *PKP2*-missense variant in a large consanguineous family with sudden death in early childhood and several members with heart transplantation in adolescent age.

## Introduction

Although multiple studies on genetics of cardiomyopathies [1–7] indicated a notable proportion of genetic cases, the genotyping of end-stage heart failure patients is not well established in clinical practice. The Human Gene Mutation Database (HGMD, [8]) lists 156 genes related to cardiomyopathies that can all necessitate heart transplantation (HTx) in the end-stage of the disease. The genes that are most commonly affected are desmosomal genes in case of arrhythmogenic right ventricular cardiomyopathy (ARVC) [9] and genes coding for components of the sarcomere, Z-disk, intercalated disk, cell membrane, ion channel, and nucleus in case of dilated cardiomyopathy (DCM), respectively [2, 4, 5, 7, 10]. Genotyping of patients evaluated for HTx, bridged to transplantation by ventricular assist device implantation (VAD) or already transplanted is still not routinely performed although this contributes to early family management [11]. Both HTx and VAD implantation may cause a considerable psychological burden for the patients and their relatives. Therefore, especially in these families a possible genetic cause of the disease is a pressing question. The knowledge of the disease-causing mutation supports the early identification of family members with asymptomatic disease or at risk for developing disease. An early patient management might prevent sudden cardiac death and might prevent or at least delay HTx or VAD implantation [11]. However, at present there are no specific guidelines available on how to treat genotype positive yet phenotype negative individuals.

The goal of this study was to evaluate whether genotyping of patients with end-stage cardiomyopathy might provide the chance to identify characteristic genes or mutations that are associated with the most severe course of this disease. Furthermore, we speculate that a notable proportion of sporadic cases are genetically determined. Therefore, we analysed 46 disease genes in 43 index-patients with end-stage cardiomyopathy by next generation sequencing (NGS). We found that the majority of DCM-cases (60%) and 40% of ARVC-cases were carriers of an at least likely pathogenic (class 4 or 5) mutation following the American College of Medical Genetics and Genomics (ACMG) guidelines for variant classification [12]. Of note, we identified a novel homozygous *PKP2*-gene mutation, which is associated with dilated cardiomyopathy.

## Material and methods

### Study population

We included patients with no-ischemic severe heart failure with need of HTx or mechanical circulatory support. We also included patients transferred to our center for the evaluation to list for HTx. A positive family history for cardiomyopathy or in case of presumed sporadic cases age at diagnosis ≤ 40 years were defined as additional study criteria. This led to the inclusion of 30 DCM (21 familial, 9 sporadic) and 3 RCM index-patients (1 familial, 2 sporadic). Additionally, for ARVC-cases an at least possible diagnosis of ARVC according the revised Task Force Criteria [13] and the modified criteria [14] in case of pediatric patients (ARVC-01 and ARVC-08) was a prerequisite. Ten ARVC index-patients (6 familial, 4 sporadic cases) met the criteria and were included in the study. Of these 4 patients had ARVC with left ventricular dilation and had been treated by HTx, and total artificial heart implantation, respectively. The remaining 6 cases were diagnosed with arrhythmias and right ventricle enlargement/failure. Four of them underwent HTx, two cases were evaluated for HTx and treated with an ICD. All patients (n = 43) were genotyped in 46 cardiomyopathy-associated genes (see below).

DCM and RCM were diagnosed according to established criteria [15, 16]. Clinical characteristics of the index-patients are summarised in Table 1, S1 and S2 Tables. All patients were of European/Caucasian origin. The written informed consent of all patients was obtained. In case of minors patient written informed consent of the parents were obtained. The study was approved by the local ethics committee (Ruhr University Bochum, Bad Oeynhausen, Germany, Reg.-No.27.1/2011). This study was complied with the Declaration of Helsinki.

**Table 1:** Clinical, familial and genetic data of 50 patients with advanced or end stage cardiomyopathy.

Patient ID	Gender	Age at diagnosis (Years)	Treatment	NYHA*/Task Force classification^†^	Age at HTx^a^/VAD^b^	Family history^‡^	Affected Genes (ACMG class)
*DCM-01*	m	1	VAD, HTx	IV	13^**a**^	Sporadic^§^	MYH7 (4), MYL2 (4)
*DCM-02*	m	31	pre HTx^II^	III	-	Sporadic	TTN (3)
*DCM-03*	m	68	ICD, VAD	III	69^b^	Son with DCM	TTN (4), DSC2 (2), TTN (3)
*DCM-04*	m	30	HTx	IV	62^**a**^	Mother (61y) died from DCM	TTN (4), DSP (3)
*DCM-05*	m	11	HTx	IV	14	Older brother with DCM and HTx	TNNC1 (4), TNNC1 (3)
*DCM-06*	m	< 1	VAD, HTx	IV	<1^**a**^	Sporadic	TTN (3)
*DCM-07*	m	35	ICD, VAD, HTx	IV	47^**a**^	Cousin with HTx, sister with suspicion of DCM, father and brother died from heart disease, sister with heart disease	LMNA (5)
*DCM-08*	m	23	VAD, HTx	IV	23^a^	Father with DCM	TTN (3)
*DCM-09*	f	41	ICD, AMB	II^#^^,^**	-	SCD of father (40y), brother died from heart disease (23y)	DES (5), TTN (3)
*DCM-10*	m	33	ICD, HTX	IV	37	Mother and aunt with HTx, brother and niece with DCM	DSP (2)
*DCM-11*	m	36	ICD, HTx	III-IV	48^**a**^	Two cousins died from SCD	DES (4)
*DCM-12*	m	18	ICD, VAD	III-IV	29^**b**^	Sporadic	TTN (4), TNNT2 (3)
*DCM-13*	m	39	pre HTx^II^	I^#^^,^**	-	Father with DCM and HTx	LMNA (4), TTN (4)
*DCM-14*	m	60	ICD, HTx	III	63^**a**^	Mother and two brothers died from heart disease, sister with DCM and HTx	RBM20 (4), DSP (3)
*DCM-15*	m	32	HTx	III	40^**a**^	Son with DCM and HTx, sister with DCM	RBM20 (5), TTN (3)
*DCM-16*	f	20	ICD, VAD	III-IV	32^**b**^	Sporadic	TTN (4), TTN (3)
*DCM-17*	m	14	ICD,HTx	III	59	Father and paternal uncle with SCD	DES (5)
*DCM-18*	f	n.a	ICD, HTx	IV	52^**a**^	Monozygotic twin sister (45y) died from heart disease	LMNA (5)
*DCM-19*	m	5	ICD, VAD	IV	22^**b**^	Sporadic	NEXN (4), MYH7 (4)
*DCM-20*	m	35	ICD, pre HTx	II-III	-	Father with DCM	TTN (4), DSP (3)
*DCM-21*	f	n.a	ICD, pre HTx	II^#^^,^**	-	Father died from DCM, deceased brother with VAD, son and daughter with heart disease	TTN (3)
*DCM-22*	f	59	pre HTx^II^	II^#^^,^**	-	Brother with HTx, another brother with VAD	TTN (4)
*DCM-23*	f	n.a.	HTx	IV	14^**a**^	SCD of 2 brothers in childhood, 2 siblings with DCM and HTx in adolescent age	PKP2 (4), LAMA4 (2)
*DCM-24*	f	30	VAD	IV	31^**b**^	Sporadic	TTN (3)
*DCM-25*	m	40	ICD, pre HTx	II-III	-	SCD of 3 maternal uncles, son with DCM	Unknown
*DCM-26*	f	n.a	HTx	IV	16^**a**^	Sporadic	Unknown
*DCM-27*	m	38	VAD, HTx	IV	53^**a**^	Father died from DCM (43y), brother and sister with heart disease	Unknown
*DCM-28*	m	37	ICD, VAD, HTx	IV	52^**a**^	SCD of maternal grandmother (60y)	Unknown
*DCM-29*	m	31	HTx	IV	42^**a**^	Paternal grandfather (51y) and father (46y) deceased from heart disease	Unknown
*DCM-30*	m	36	ICD, pre HTx	II**	-	Sporadic	Unknown
*RCM-01*	f	12	VAD, HTx	IV	13^**a**^	Sporadic	MYL3 (3)
*RCM-02*	m	< 1	VAD	IV	<1^**b**^	Sporadic	TNNI3 (4)
*RCM-03*	m	19	HTx	IV	12^**a**^	Mother (42y) with muscular dystrophy died from heart disease	CRYAB (5)
*ARVC-01*	m	14	ICD	DD^††^**		Sporadic	PKP2 (5)
*ARVC-02*	m	35	ICD, HTx	DD	36^**a**^	SCD of father, brother with heart disease	MYH7 (3)
*ARVC-03*	m	67	ICD	PD		Sporadic	PRKAG2 (3)
*ARVC-04*	f	35	ICD, TAH	DD	55^**b**^	SCD of maternal grandmother and uncle, sister with DCM	PLN (5), MYH6 (3), TTN (2)
*ARVC-05*	f	45	ICD, HTx	DD	47^**a**^	Maternal grandfather with SCD	LMNA (4), PKP2 (3)
*ARVC-06*	f	21	ICD, HTx	DD	32^**a**^	ARVC with SCD of father and brother	PKP2 (5), PKP2 (3), RYR2 (3), TTN (3)
*ARVC-07*	m	40	ICD, HTx	BL	62^**a**^	Paternal great-grandfather and father with heart disease and premature death	DES (3)
*ARVC-08*	m	16	ICD, HTx	BL^**††**^	21^**a**^	Sporadic	Unknown
*ARVC-09*	f	42	VAD	PD	45^**b**^	Sporadic	Unknown
*ARVC-10*	f	n.a	ICD, HTx	DD	46^**a**^	Two maternal great-uncles with SCD	Unknown

Abbreviations

**a** = age at HTx, **ACMG class 2** = likely benign, **ACMG class 3** = variant of uncertain significance, **ACMG class 4** = likely pathogenic, **ACMG class 5** = pathogenic, **AMB** = ambulatory, **ARVC** = arrhythmogenic right ventricular cardiomyopathy

**b** = age at VAD, **DCM** = dilated cardiomyopathy; **f** = female, **FS** = fractional shortening, **HTx** = heart transplantation, **ICD** = implantable cardioverter defibrillator, **ID** = identification, **m** = male, **n.a.** = not available, **RCM** = restrictive cardiomyopathy, **SCD** = sudden cardiac death, **TAH** = total artificial heart, **VAD** = ventricular assist device; **y** = year(s).

^*^New York Heart Association functional classification of heart failure before VAD or HTx, respectively.

^**†**^Classification according Task Force Criteria [13].

^**‡**^Familial disposition for cardiomyopathy due to pedigree analysis and anecdotal evidence.

^§^Sporadic = isolated cases without family history.

^II^Treated medically.

^**#**^Progressive cardiomyopathy with familial disposition due to pedigree analysis or anecdotal evidence, respectively.

**Not yet listed for HTx but in the long term surveillance program.

^**††**^ Classification according to Task Force Criteria [13] and consideration of the modified pediatric criteria [14]. For pedigrees see S2 Fig.

### DNA analysis

DNA was isolated from white blood cells using standard techniques (High Pure PCR Template Preparation Kit^®^, Roche Diagnostics GmbH, Mannheim, Germany) and prepared for cardiac gene enrichment re-sequencing on a MiSeq^®^ next generation sequencing system according to manufactures instructions (TruSight™ Rapid Capture Sample Preparation Kit). All patients were screened for variants in 46 cardiomyopathy genes using the TruSight™ Cardiomyopathy gene panel. The evidence linking these genes to cardiomyopathy has been published previously [17] (for gene list see S3 Table). Enrichment, sequencing kits and sequencing system were supplied by Illumina Inc, San Diego, CA, USA. For reads alignment and base calling Illumina´s automated work flow was used (BWA [18], GATK [19], and SAMtools [20]).

A minimum read depth of ≥ 20 reads was set as quality cut-off (quality data see S4 Table). For variant annotation the VariantStudio™ v2.1 software provided by Illumina Inc with the UCSC Genome Build hg19 as reference was used. We filtered variants located in exon- and consensus splice-sequences (± 1 and 2 position) with a global minor allele frequency (MAF) ≤0.0005 according to the data provided by the Exome Aggregation Consortium (Version 0.3, ExAC data and browser (beta) Version) [21]. Synonymous variants were not taken into account. Variants of interest were verified by Sanger sequencing (BigDye^®^ Terminator v1.1 Cycle Sequencing Kit, ABI PRISM^®^ 3100 genetic analyzer, Applied Biosystems, Foster City, CA, USA).

### Variant classification

This study was primarily designed to identify dominant single gene mutations with clear clinical impact on ARVC or other cardiomyopathy subtypes. Therefore the guidelines [12] of the American College of Medical Genetics and Genomics (ACMG) were considered for variant classification. The ACMG defined five variant classes: class 5, pathogenic; class 4, likely pathogenic; class 3, variant of uncertain significance (VUS); class 2, likely benign; class 1, benign. For use on mainly private variants the criteria and rules for classification have been adjusted (for details see S1 Methods). The cut-off for the minor allele frequency (MAF) of variants of interest was set to a value ≤0.0005 and thus within the range of the respective disease prevalence (http://www.orpha.net) [22]. The ExAC browser [21] was used as reference for allele frequencies. Only frequencies of the non-Finnish European population were taken into account. Original reports listed in the Human Mutation Database Professional 2015.1 (HGMD^®^) [8] and the ARVC Genetic Variants Database [23, 24] were reviewed concerning variant classification. Deleterious effect of a variant was considered when ≥3 prediction tools forecasted the variant to be deleterious (MutPred [25], SNP&GO [26, 27], Fathmm [28], and PolyPhen-2 (Polymorphism Phenotyping v2, humVar) [29]). In case of the *TTN*-gene only variants affecting the cardiac titin isoforms N2B and/or N2BA were further investigated. In this manuscript only (likely) pathogenic gene variations are referred to as ‘mutation’ while (likely) benign variants, variants of uncertain significance and gene variations in a general context are called ‘variant’.

### Gene and variant nomenclature

The nomenclature of sequence variants based on the rules of the Human Genome Variation Society (HGVS, www.hgvs.org). Nucleotide and amino acid numbering refers to the reference sequences given by the National Center of Biotechnology Information (NCBI, http://www.ncbi.nlm.nih.gov) (S3 Table). In case of *TTN* the nomenclature refers to the longest predicted isoform (NM_001267550.1, NP_001254479.1). All variations listed in this manuscript were submitted to ClinVar (http://www.ncbi.nlm.nih.gov/clinvar) using the submission name HDZNRW. An already existing listing of the study variants in the ClinVar database was checked. Some of them have been reported by others, a few with conflicting interpretation of pathogenicity. The listing of *TTN* variants was only partially verified because of inconsistent nomenclature based on different isoforms.

## Results

### Patient cohort

**Fig 1:**
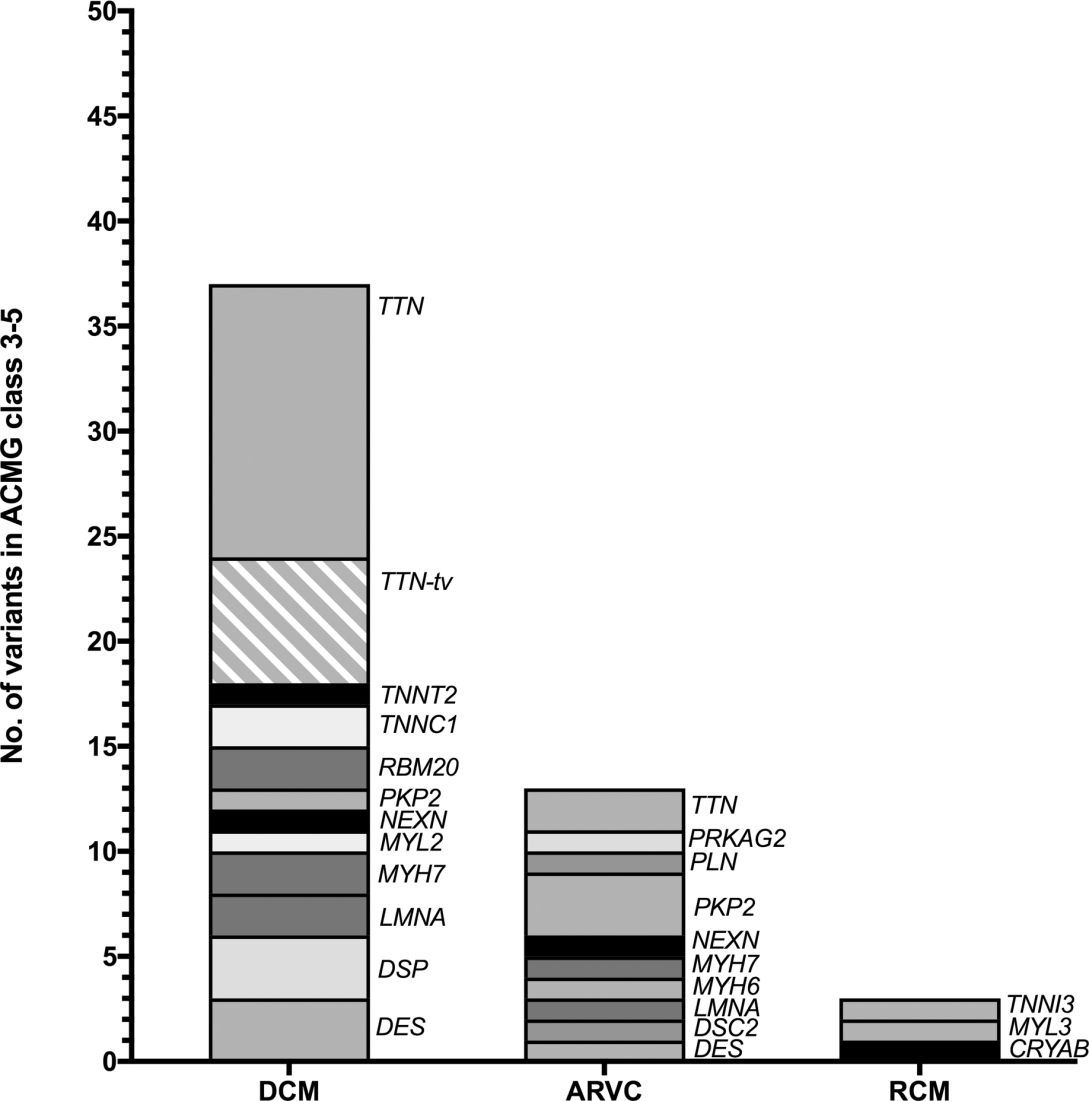
Variant carrying genes. In DCM-cases *TTN* carried the most variants. Of 19 *TTN*-variants in DCM-cases, 6 were truncating variants. The other affected genes were *TNNT2*, *TNNC1*, *RBM20*, *PKP2*, *NEXN*, *MYL2*, *MYH7*, *LMNA*, *DSP*, and *DES*. In ARVC-cases predominantly *PKP2*- and *TTN*-variants were identified. Further variants were found in the genes *PRKAG2*, *PLN*, *MYH7*, *MYH6*, *LMNA*, and *DES*. In the cohort of the RCM-cases variants in 3 different genes were identified: *TNNI3*, *MYL3*, and *CRYAB*. ACMG class 3–5 *=* variant of uncertain significance, likely pathogenic, pathogenic, respectively.

The study cohort comprised 43 unrelated index-patients (29 male, 14 female) with cardiomyopathy of different types (Fig 1). Of these, 33 patients presented with end-stage disease and already underwent implantation of VAD and/or HTx. Ten patients were evaluated for HTx due to clinical signs of advanced cardiomyopathy. Seventy-nine percent of DCM- and RCM-patients were in New York Heart Association (NYHA) classification III or IV (Table 1). Six ARVC-patients were classified with definite diagnosis (DD), 2 patients with possible (PD), and 2 patients with borderline diagnosis (BL) (Table 1). The mean age at diagnosis was not significantly different in DCM- (0031±17 years) and ARVC-cases (35±17 years) (S1 Fig). Due to the small number statistical evaluations for RCM-cases were not possible in this study (Table 1). Familial disposition for the cardiomyopathy due to pedigree analysis and anecdotal evidence was suspected in 21of 30 DCM, 6 of 10 ARVC and once in RCM (n = 3) cases (Table 1). Sporadic cardiomyopathy was suspected in 15 patients. Where available the pedigrees illustrating heart disease within the families and variant co-segregation/penetrance are shown in S2 Fig.

### Genotyping results

**Fig 2:**
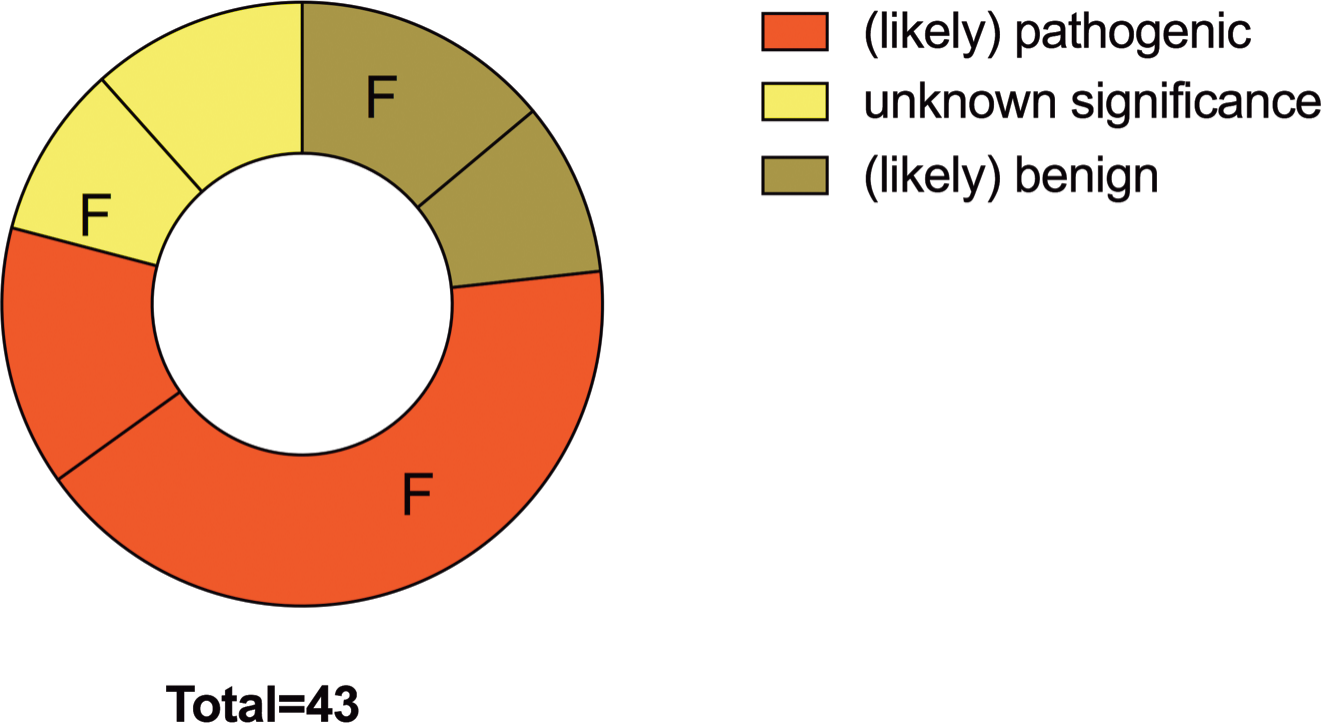
Distribution of variant classes. We genotyped 43 index-patients. Of these 18 familial and 6 sporadic cases carried (likely) pathogenic mutations. At least variants of uncertain significance were found in 4 familial and 5 sporadic cases. Only likely benign variants or variants with an allele frequency exceeding the disease prevalence were identified in 10 patients (6 familial, 4 sporadic cases). Familial disposition for the cardiomyopathy is based on pedigree analysis and family reports. **Abbreviations: F** = familial cases, **no label** = sporadic cases.

In the entire study population we identified (likely) pathogenic mutations in 56% of index-patients (18 familial, 6 sporadic cases). Nine patients were carriers of a pathogenic and 15 carried a likely pathogenic mutation. VUS were found in 21% of cases (4 familial, 5 sporadic cases). An only likely benign variant was identified in one patient and variants with an allele frequency exceeding the disease prevalence and therefore not further investigated were identified in 9 patients. This means that in total in 23% (n = 10, 6 familial, 4 sporadic cases) of patients no disease associated variants were identified (Fig 2).

**Fig 3:**
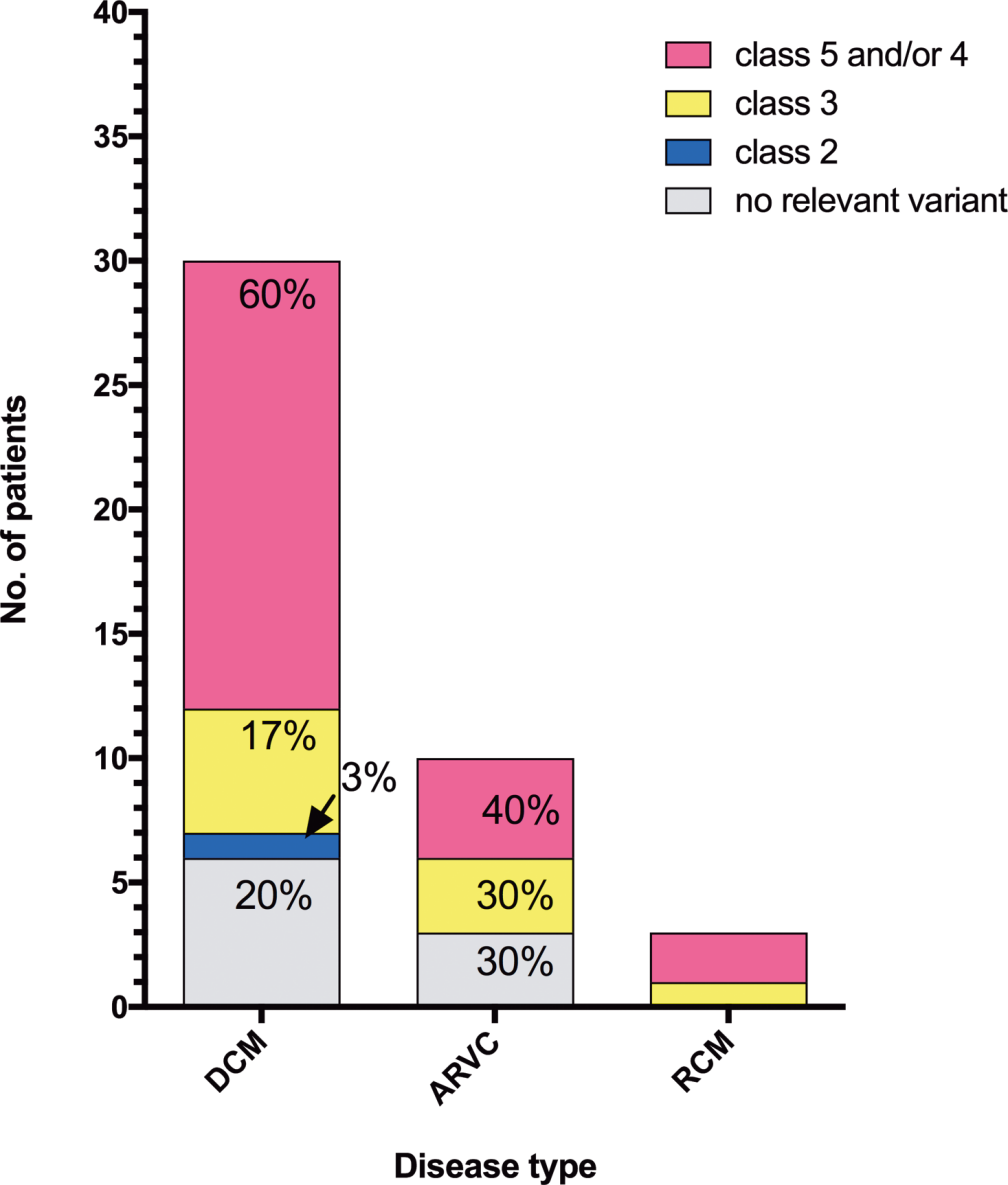
Classification of the variants. In 60% of DCM- and 40% of ARVC-cases pathogenic and/or likely pathogenic mutations were identified. At least variants with uncertain significance were found in 17% DCM- and 30% ARVC-cases. No relevant variants (MAF >0.0005 in etiological matching controls) were found in 22% and 30% of DCM-, and ARVC-cases, respectively. In RCM-cases, 2 pathogenic and/or likely pathogenic mutations and 1 variant with uncertain significance were identified. **Abbreviations**: **class 2** = likely benign, c**lass 3** = variant of uncertain significance, **class 4** = likely pathogenic, **class 5** = pathogenic.

Of the DCM-patients, 60% (n = 18) had pathogenic or likely pathogenic mutations, respectively, while 17% (n = 5) were at least carriers of VUS. One DCM-case (DCM-10) had only a likely benign variant. In DCM-patients 25–30 only polymorphisms were identified (Fig 3). The proportion of pathogenic or likely pathogenic mutations was 66% in familial DCM and 44% in sporadic DCM-cases. In 40% (n = 4; 3 familial, 1 sporadic case) of ARVC-cases pathogenic or likely pathogenic mutations were found (Fig 3). In the entire ARVC-cohort (likely) pathogenic mutations were identified in 50% of familial cases (n = 6). In sporadic cases (n = 4) one pathogenic mutation was identified. Three ARVC-patients each were carriers of VUS or polymorphisms, respectively (Fig 3). In the RCM-cases (n = 3) two patients with pathogenic or likely pathogenic mutations (1 familial, 1 sporadic case), respectively, were identified (Fig 3).

### Variant classification

In 19 of 46 genes, a total of 57 variants were found (Fig 1). We identified 41 missense variants in addition to 3 nonsense, 7 frameshift, 3 in frame insertion/deletion, and 3 canonical splice-site variants. Out of these, 15 had entries in the mutation-databases [8, 24], whereas 42 variants were previously not described in context with a disease. We evaluated the pathogenic evidence of all variants (S5 and S6 Tables) and classified four variants likely benign, whereas fifty-three variants were classified (likely) pathogenic or VUS, respectively. The potentially disease related variants affected 18 genes. In total, we identified 9 pathogenic mutations in 9 index-patients (8 familial cases, 1 sporadic case) in 6 different genes (*LMNA*, *DES*, *RBM20*, *CRYAB*, *PKP2*, *PLN*). We identified two or more variants in 14 DCM- and 3 ARVC-cases.

In DCM-patients *TTN* was the most frequently affected gene (51% of variants; Fig 1). In the DCM-cohort, pathogenic mutations were identified in *DES*, *LMNA*, and *RBM20* (Table 1, S5 Table), whereas likely pathogenic mutations were found in *MYL2*, *MYH7*, *TTN*, *TNNC1*, *DES*, *LMNA*, *RBM20*, *NEXN*, and *PKP2*. Remarkably, a homozygous *PKP2-*gene mutation (c.2035C>T, p.His679Tyr) was identified in a large Turkish family (DCM-23). As expected, *PKP2* was the most frequently affected gene in ARVC-patients. Pathogenic mutations in the ARVC-cohort were found in *PKP2* and *PLN*, a likely pathogenic mutation was found in *LMNA* (Table 1, S5 Table). Surprisingly, no further desmosomal genes were affected in ARVC-cases. In the RCM-cases two patients with pathogenic or likely pathogenic mutations in *TNNI3* and *CRYAB*, respectively, were identified (Table 1, S5 Table). The median age at diagnosis in DCM- and ARVC-cases (33 and 36 years, respectively) was not significantly different in dependence of variant classification (S3 Fig).

Twenty-one *TTN* variants (7 *TTN*-tv (premature stop codon with/without preceded frameshift, and canonical ± 1 or 2 splice site variants), 13 missense, 1 in-frame insertion) were identified in 16 (37%) of 43 index-patients (Table 1, S5 Table). In DCM-patients *TTN*-variants were more frequent (p<0.05; S4 Fig), whereas *TTN*-tv were increased by trend (p = 0.05, data not shown) when compared to the other cardiomyopathies. Most of the *TTN*-variants (5 *TTN*-tv, 7 missense) affected the titin domains spanning the A-band of the sarcomere. But also variants in the I-band (3x missense, 2x *TTN*-tv), M-line (2x missense, 1x in frame insertion), and Z-band (1x missense) region of the protein were identified (S7 Table). All but one *TTN*-variant were located within exons that are estimated to be incorporated in 100% of the cardiac *TTN*-transcripts (PSI 100%, [6], http://cardiodb.org/titin) and might be expressed to a notable amount. The *TTN*-variant found in patient DCM-2 is located within the I-band and is estimated to be expressed only in 8% of the transcripts and is therefore believed to be of low disease relevance [6]. Seven (21%) of the 34 variant carriers of the present study had only *TTN*-variant(s). Of these four patients had a single *TTN* missense variant affecting the I-, A, and Z-band of titin, respectively. One patient was carrier of a single *TTN*-tv in the A-band of the protein. DCM-16 had a splice and a missense *TTN*-variant in the A- and I-band, respectively. DCM-24 is carrier of one in-frame duplication and two missense variants affecting the M-line and the A-band, respectively (S5 and S7 Tables).

### Family with a novel homozygous *PKP2*-gene mutation

**Fig 4:**
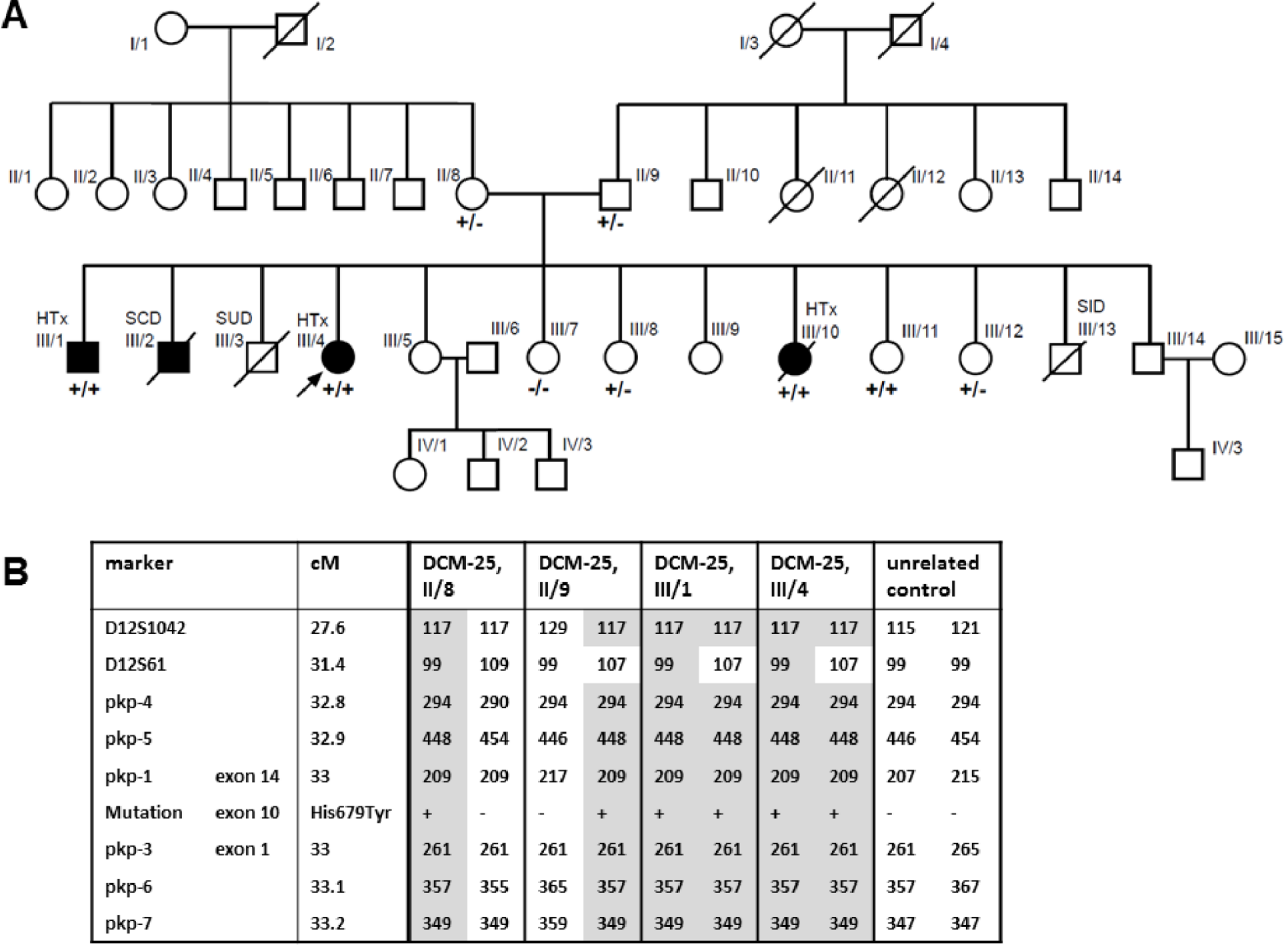
Pedigree of DCM-23 family and PKP2 c.2035C>T co-segregation. **A.** Family members with HTx were homozygous carriers of *PKP2* c.2035C>T, p.His679Tyr whereas heterozygous carriers and the homozygous carrier III/11 had no signs of cardiomyopathy. The *LAMA4* variant did not co-segregate with disease (data not shown). Squares = males, circles = females. Deceased individuals are indicated by slashes. Filled symbols indicate individuals with DCM. The index-patient is marked with an arrow. Genotypes are shown by present (+) or by absent (-) of the gene variant. **Abbreviations**: **SCD** = sudden cardiac death, **SID** = sudden infant death, **SUD** = unexplained sudden death. **B.** Haplotype analyses of *PKP2* c.2035C>T mutation carriers from family DCM-23. Haplotypes associated with the *PKP2* mutation are shown in grey shaded areas. These haplotypes are identical in both parents (II/8, II/9) with the exception of marker D12S61. Abbreviation: **cM** = centimorgan.

We identified a novel homozygous *PKP2*-mutation c.2035C>T, p.His679Tyr in index-patient DCM-23 (III/4, Fig 4A) who is a member of a large non-consanguineous Yezidi family. The affected family members did not fulfill the ARVC Task Force criteria although *PKP2* is generally associated with ARVC. Three genetically untested brothers died suddenly in childhood (III/2, III/3, III/13; 11 years, 2 years, 7 days, respectively). She and two siblings (male (III/1), female (III/10)) who are also homozygous for the *PKP2*-mutation underwent HTx in adolescent age (14, 13 and 17 years, respectively) because of DCM (ECGs shown in S5 Fig.). The 57 years old mother (II/8), the 66 years old father (II/9) and two other siblings (III/8, III/12) who are heterozygous mutation carriers have no signs of cardiac disease at all, suggesting a variant co-segregation in an autosomal recessive mode. Surprisingly, the youngest sister (III/11, 18 years old) who is also a homozygous mutation carrier has currently no detectable signs of cardiomyopathy as examined by ECG, TTE and MRI (data not shown). The suspected pathogenic impact of *PKP2* c.2035C>T was verified by analyzing whole exome data of the parents, an unaffected sister (III/8), and the index-patient (III/4) (S1 Methods, S6 Fig). No other co-segregating variant with an allele frequency ≤0.0005 was found in cardiomyopathy genes listed in HGMD (n = 156, S8 Table). A further indication of pathogenicity is the absence of *PKP2* c.2035C>T in the ExAc browser with sufficient coverage of the gene region (read depth of 50 in 80% of the alleles). Analysis of the *PKP2* locus (Fig 4B, S1 Methods) of the index-patient (III/4) and her transplanted brother (III/1) revealed that they are homozygous for a haplotype surrounding the *PKP2*-gene which largely overlaps between father (II/9) and mother (II/8), suggesting that they are probably (distantly) related and the mutation might be inherited from a common ancestor. In order to study the pathogenic mechanism of the mutation we investigated plakophilin-2 mRNA and protein levels in explanted myocardium, but no differences compared to controls were detected (S1 Methods, S7 and S8 Figs). Immunofluorescence staining of myocardial sections of the explanted heart of individual III/10 showed normal localization of PKP2 at the intercalated disc (S1 Methods, S9 Fig). Thus, the mutation appears to have no influence on the stability or subcellular localization of PKP2 within the myocardium. No segregation with disease was observed for the nonsense variant in *LAMA4* (S5 Table).

## Discussion

This study focused on the genotyping of patients with advanced cardiomyopathy. Our inclusion criteria lead to a study cohort mainly composed of patients who have been bridged for heart transplantation and those already transplanted. The study design was set for the identification of highly penetrant monogenic causes of cardiomyopathies.

In total 57 variants were identified of which a high proportion were classified (likely) pathogenic (51%). Surprisingly, (likely) pathogenic mutations were more frequently present in DCM- than in ARVC-cases (60% versus 40%) although the majority of currently known ARVC-genes with the exception of the minor disease genes *TGFB3*, *CTNNA3* (see www.arvcdatabase.info), [4], *FLNC* [30], and *SCN5A* [31] were analyzed. No variants were identified even in one definite and one familial ARVC-case. This strongly suggests that other currently unknown genes and/or genotypes are involved in the development of ARVC. Especially future screening of the *FLNC*-gene could increase the variant detection because a prevalence of 1% of truncating *FLNC*-mutations was reported in dilated and arrhythmic cardiomyopathies [30].

The mutation rate and profile in ARVC patients is in discrepancy to what have been expected and published before by others. For example Groeneweg et al. [32] found a mutation rate of 63% in a large ARVC-cohort, and in desmosomal genes a mutation rate of 40% was reported [33]. The high mutation rate in our DCM-cohort is in the upper range of values reported so far [2, 4, 5], in case of familial DCM it is in agreement with the findings of Cuenca et al. [7]. Apparently, the mutation profiles differ notably between the different studies. As expected, screening for gene variants is more successful in familial cases, but also in presumably sporadic cases (likely) pathogenic mutations were identified in a noteworthy high number (25%). In previous studies the prevalence of (potentially) pathogenic mutations in sporadic DCM-cases was 8% and 68%, respectively [4, 5]. Our study cohort includes 12 patients with disease onset at ≤20 years of age assuming a genetic cause of disease although familial cardiomyopathy was reported only for four cases. In eight patients an at least likely pathogenic gene mutation was identified. These findings underscore the relevance to perform genetic testing not only in suspected familial or sporadic cases with clinical clues suggestive of a particular/rare genetic disease as recommended by the European Society of Cardiology [11] but in all index-patients with an unknown etiology of the disease. Nevertheless, a characteristic mutation profile for severe cardiomyopathy could not be defined.

Twenty five percent of our DCM cohort carried a *TTN*-tv mutation as reported for end-stage DCM patients by Roberts et al. [6]. All *TTN*-tv mutations were classified as likely pathogenic considering a high prevalence of *TTN*-tv in DCM-cohorts. *TTN* alternative splicing, position-independent nonsense-mediated mRNA decay, an internal promoter, allele-specific expression, structural and functional effects on sarcomere formation and force generation as well as negative effects by incorporation of truncated titin into the sarcomere were discussed as pathomechanisms for truncated titin [34–39]. Nevertheless, many additional data are required for a better understanding of the mechanisms. Because of the background noise in the general population *TTN* missense variants were classified as VUS although recent large-scale sequencing and functional analysis indicated strong impact on cardiomyopathies [40–42]. Protein unfolding and domain destabilization was shown and/or predicted for *TTN* missense mutations located in the Z-disk [42], A/I junction region [43], and in the I-band [41] of the protein, respectively. Decreased contractile force generation of a missense mutation located in the Z/I-band junction was reported by Hinson et al. [35]. We identified 7 index-patients with only *TTN* variants of different types affecting different protein regions. But although functional data are available a final classification of *TTN* variants is not possible. Classification still based mostly on populations genetic data and one should keep in mind that new functional data could lead to variant re-classification. Two patients carried multiple *TTN* variants. We did not investigate whether the gene was affected in a compound heterozygous or homozygous way. Regardless, no data are available about patients with several *TTN* variants and about a possibly increasing impact on disease. In agreement with recent data [6, 40] most *TTN*-variants affected the inextensible A-band of titin and are incorporated in 100% of the cardiac *TTN*-transcripts. [44, 45]. Patient DCM-16 is carrier of a *TTN*-tv and missense variant, had disease onset at the age of 20 years and VAD implantation when she was 32 years old. Therefore, we suggest that *TTN* associated cardiomyopathy is present also in young adults even though mostly it has its onset in the mid-late life period [40].

We identified a non-synonymous *PKP2*-mutation that in homozygous carriers led to manifestation of end-stage DCM at young age. This mutation might be characterized by a reduced penetrance [46–48] since at present a homozygous 18 years old mutation carrier has no signs of cardiac disease. We cannot rule out additional genetic factor(s) underlying the disease of the family, although screening of all known cardiomyopathy associated genes gave no indication for this. For *PKP2* truncating mutations and interestingly, also for missense mutations haploinsufficiency was indicated as a possible pathomechanism. Functional data to support this assumption are limited. Kirchner et al. [49] reported decreased *in vivo* and *in vitro* protein expression, impairment of protein structure and stability, and mislocation of plakophilin 2 in the cytoplasm in case of *PKP2* missense mutations. In addition for other *PKP2* missense mutations impairment of *in vitro de novo* desmosome assembly was described [50] and influence on the expression of heat shock protein 90 was observed in case of two *PKP2* truncating mutations [51]. In our study the investigation of heart tissue of a homozygous mutation carrier revealed no impact of *PKP2* p.His679Tyr on plakophilin expression, stability and cell localization. This might indicate a different pathomechanism as described above, although it must clearly be pointed out, that in contrast to our *in vivo* data the characteristics of the mutations described by Kirchner et al. based mainly on cell culture experiments. To our knowledge, this is the first report of a *PKP2*-missense mutation causing recessively inherited DCM. No signs of hair or skin abnormalities that were reported with other recessive desmosomal gene mutations [52, 53] were observed.

We detected a missense mutation in the Z-disk protein NEXN that was already described to have a disruptive effect on the cardiac sarcomere in a DCM patient and a dominant negative effect on Z-disk stability in zebrafish [54]. In accordance, we found this mutation in a male DCM patient (DCM-19) who underwent VAD implantation at the age of 22 years. Based on the functional studies described by Hassel et al. [54], an allele frequency clearly below the prevalence of DCM, and multiple computational evidence for a deleterious effect on the protein this *NEXN* mutation must be classified as likely pathogenic according to ACMG guidelines. Our study is the second report that links the *NEXN* gene to DCM and thereby strengthens the importance of *NEXN* as a (minor) disease gene.

In our study, the usage of a sequencing panel with a comparatively small number of myopathy associated genes for genotyping patients has historical reasons. But despite the limited gene number it is important to point out that the majority of variants identified here were novel variants in known major disease genes which are listed in the Mendelian Inheritance in Man (MIM) compendium and which are covered by the 46-gene panel used here. As expected besides these also variants in minor disease genes were identified.

Extensive variation screening in other (minor) disease genes in the whole exome and genome, respectively, might provide further insights. However, variant classification with respect to the impact on disease development and clinical course still remains the real challenge [12]. Due to limited availability of case reports, small families with limited co-segregation data, and rarely available functional data, some ACMG [12] criteria were not applicable and others had to be adjusted. But apart from this limitation, the ACMG guidelines were a suitable tool for a uniform and differentiated variant classification. At present there are no guidelines available how to treat non-symptomatic mutation carriers, due to the heterogeneous genetic background of cardiomyopathies and the lack of pharmacological evidence for preventive treatment. In order to overcome this limitation in the future more studies like the recent trials that investigate the effect of drug treatment of pre-symptomatic *PLN* and *LMNA* mutation carriers are of impact (Clinical trials identifier: NCT01857856 and European Society of Cardiology Congress 2016, Rome, Poster P4981, respectively).

## Limitation

We excluded variants with an allele frequency above the disease prevalence. Therefore, this study is not suited for the identification of a complex genotype or modifiers as well as variants with reduced penetrance. Copy number variants and large DNA-rearrangements have not been investigated. Only allele frequencies of the ethnically narrow non-Finnish European population provided by the Exome Aggregation Consortium were taken into account to define variants of further interest. Variant screening was limited to 46 myopathy associated genes covered by the TruSight™ Cardiomyopathy gene panel of Illumina. The recently described ARVC and DCM disease gene *FLNC* and the ARVC disease gene *SCN5A* were not screened for variants.

## Supporting information

S1 Methods Supplementary methods. (DOCX)

S1 Table Clinical data and family history. (DOCX)

S2 Table Task Force classification and family history of ARVC index-patients. (DOCX)

S3 Table Illumina myopathy panel 46 gene list. (DOCX)

S4 Table 46-gene panel next generation sequencing quality data. (DOCX)

S5 Table Overview on genotyping results and classification according to ACMG guidelines. (DOCX)

S6 Table Considered criteria for variant classification according to ACMG guidelines. (DOCX)

S7 Table *TTN*-gene variants. (DOCX)

S8 Table Genes listed in HGMD when using the search term `cardiomyopathy`. (DOCX)

S1 Fig Age of patients at the initial diagnosis. (DOCX)

S2 Fig Family histories and variant co-segregation. (DOCX)

S3 Fig Age of patients at the initial diagnosis vs variant classification. (DOCX)

S4 Fig Number of *TTN* variants in DCM patients compared to other cardiomyopathy. (DOCX)

S5 Fig Electrocardiogram of patients III/1 and III/10 of family DCM-23. (DOCX)

S6 Fig Quality score distribution of whole exome sequencing data of family DCM-23. (DOCX)

S7 Fig Real-time reverse transcription PCR of *PKP2*-mRNA. (DOCX)

S8 Fig Western blot against PKP2 and GAPDH. (DOCX)

S9 Fig Immunofluorescence staining of myocardial sections using confocal microscopy. (DOCX)
