# Supplementary material for: High proportion of genetic cases in patients with advanced cardiomyopathy including a novel homozygous Plakophilin 2-gene mutation
Source: PLoS One. 2017 Dec 18;12(12):e0189489. doi: 10.1371/journal.pone.0189489 (PMC5734774; doi:10.1371/journal.pone.0189489)
Supplement: S1 Methods — (DOCX) [file pone.0189489.s001.docx]

**S1 Methods**

**Variant classification**

The recently published guideline for variant classification of the American College of Medical Genetics and Genomics (ACMG) were considered ([1](#_ENREF_1)). The ACMG defined 5 variant classes: class 5, *pathogenic*; class 4, *likely pathogenic*; class 3, *uncertain significance*; class 2, *likely benign*; class 1, *benign*. For use on mainly private variants the criteria and rules for classification have been adjusted:

In case of the ACMG criterion PM2 (“Absent from controls or at extremely low frequency”, for details see ([1](#_ENREF_1))) the cut off for the MAF of variants of interest was set to a value ≤ 0.0005. Thus, the cut off is within the range of the respective disease prevalence (DCM: 0.0001-0.0005, ARVC: 0.0001-0.0005, RCM: 0.00001-0.00009 (http://www.orpha.net), LVNC: <0.0005 ([2](#_ENREF_2)), for the only HCM case in this study the cutoff was set to 0.0005 since the patient fulfilled the criteria of a HNOCM, which has a different prevalence ([3](#_ENREF_3)) as compared to HCM in general. The ExAC browser (Beta, Version 0.3, ([4](#_ENREF_4)), data of more than 60,000 unrelated individuals e.g. from the 1000 Genomes project ([5](#_ENREF_5)) and the NHLBI-GO Exome Sequencing Project (ESP) ([6](#_ENREF_6))([6](#_ENREF_6))) was used as a reference data set of allele frequencies for the variants under investigation. Only frequencies of the non-Finnish European population were taken into account (etiologically matching control group). Currently this is the most comprehensive data set of more than 60,000 unrelated individuals including data e.g. from the 1000 Genomes project ([5](#_ENREF_5)) and the NHLBI-GO Exome Sequencing Project (ESP) ([6](#_ENREF_6))([6](#_ENREF_6)). ACMG criterion BS4 (“Lack of segregation in affected members of a family”, for details see ([1](#_ENREF_1))) was used as *stand-alone* criterion with subsequent downgrading of class 3 variants to class 2. The Human Mutation Database (HGMD^®^) Professional 2015.1 ([7](#_ENREF_7)) and the ARVD/C Genetic Variants Database ([8](#_ENREF_8), [9](#_ENREF_9)) were used to verify known variants. The original reports were studied and the variants were re-classified according to the ACMG guidelines. Computational evidence of deleterious effects were determined by the use of 4 types of prediction tools that are previously described to give the best results ([10-12](#_ENREF_10)): (1) MutPred ([13](#_ENREF_13)), (2) SNP&GO ([14](#_ENREF_14), [15](#_ENREF_15)), (3) Fathmm ([16](#_ENREF_16)), and (4) PolyPhen-2 (Polymorphism Phenotyping v2, humVar) ([17](#_ENREF_17)). The requirement for the ACMG criterion PP3 (“Multiple lines of computational evidence support a deleterious effect,” for details see ([1](#_ENREF_1))) was considered fulfilled if ≥3 prediction tools forecasted the variant to be deleterious. MutPred was not available for large data volumes. For this reason substitutions within the *TTN-*gene were analysed with the remaining prediction tools. Computational analysis was performed only for missense variants. Positive functional data (ACMG criterion PS3, for details see ([1](#_ENREF_1))) were defined as any investigation of *in vivo* or *in vitro* models demonstrating effects differing from wild type and matching the disease phenotype. In case of the *TTN*-gene only variants affecting the cardiac titin isoforms N2B and/or N2BA were further investigated. Titin-truncating variants (*TTN*-tv: premature stop codon with/without preceded frameshift, and canonical ± 1 or 2 splice site variants) of present study were classified as class 4 variants instead of class 5 taken into account that *TTN*-tvs were also identified in healthy controls although less frequent than in DCM-cohorts ([18](#_ENREF_18), [19](#_ENREF_19)). In this manuscript class 4-5 variants are called ‘mutations’, class 1-3 variants are called ‘variants’.

**Isolation of total RNA and Real-time quantitative PCR with TaqMan® probes**

Total RNA was isolated from left heart ventricles provided after heart transplantation using commercial kits (RNeasy, Qiagen, Hilden, Germany) as previously reported ([20](#_ENREF_20)). For RNA isolation about 30 mg myocardial tissue was used. Purified total RNA was quantified photometrically at 260 nm and RNA-purity and -integrity were assessed by agarose-gel electrophoresis.

Reverse transcription of myocardial RNA was performed using 250 ng total RNA and 50 units of the enzyme Superscript II (Invitrogen, Netherlands) after random priming with hexamers. Using 2 µl of the reverse reaction as a template we quantified the mRNA of plakophilin 2 (PKP2) using glycerinaldehyde-phosphate-dehydrogenase (GAPDH) as a housekeeping gene on a StepOnePlus™ real-time PCR system (Applied Biosystems, Foster City, CA, USA) performing duplicates. Relative quantification was evaluated using the comparative C_T_-method (ΔΔC_T_) of the StepOne^TM^ (v2.0) software. Primer sequences are available from the authors upon request. The conditions for the PCR-reaction were: 95°C, 10 min for initial denaturation, 40 cycles 60°C, 1 min/ 95°C, 15 sec using TaqMan® universal master mix (Applied Biosystems, Foster City, CA, USA).

**Tissue-immunohistochemistry**

Immunohistochemistry was performed on 5 µm thick paraffin embedded longitudinal sections of left ventricular heart muscle tissue provided after HTx and of a rejected donor heart, respectively. Following primary antibodies were used in this study: (1) mouse monoclonal anti-PKP2 antibody (dilution 1:500; #ab151402, Abcam, Germany) and (2) anti-connexin 43/GJA1 antibody (dilution 1:1000; #ab11370, Abcam, Germany). As secondary antibodies (1) rat monoclonal secondary antibody to mouse IgG FITC (dilution1:100, #ab133859, Abcam, Germany) and (2) sheep anti-rabbit IgG Cy3 F(ab)^2^ fragment (dilution 1:500, #C23076, Sigma, Germany) were used. Immunostaining was performed according to the recommendations of the manufacturers. Fluorescence labeling was evaluated with an inverted microscope (NIKON Eclipse, TE 2000 U, NIKON, Germany).

**Westernblotting**

30 mg heart muscle tissue provided after HTx was homogenized in 300 µl lysis buffer (30 mM Tris base, 2 M thiourea, 7 M urea, 4 % CHAPS, pH 8,5). Mechanical cell lysis was performed by ultrasonic treatment for 1 minute on ice. Protein concentration in the supernatant (16000 g for 15 minutes at 4 °C) was determined by the Bradford method. Of each sample 10 µg protein was diluted in loading buffer (125 mM Tris, 4% (v/w) SDS, 20 % (v/v) glycerol; 1 % (v/v) ß-mercaptoethanol, bromphenolblue, pH 7,0) and used for electrophoresis. Electrophoresis was performed on a 10 % SDS-PAGE gel, followed by the transfer to a nitrocellulose membrane and blocking with 5 % non-fat dry milk in TTBS (0,02 M Tris, 0,1 M NaCl, pH  7,5, 0,1% Tween) for 30 minutes. The membrane was incubated with the primary antibody for 12 hours (α-PKP2: ProGen, Heidelberg, Germany; α-GAPDH: abcam, Cambridge, England) followed by the incubation with the secondary HRP-linked antibody for 1 hour (Biosciences Pharmingen, New Jersey, USA). An enhanced chemiluminescence detection system (Advansta, Menlo Park, USA) was used to visualize immunoreactive bands. The concurrent detection of glycerinaldehyde-phosphate-dehydrogenase (GAPDH) was used as loading control. A MultiImage™ Light Cabinet (Biozym Diagnostics GmbH, Germany) and the FluorChem FC2 V3.2.2 software (Cell Bioscience Inc.) were used for documentation and band analysis, respectively.

**Haplotype Analysis**

To determine whether the homozygous *PKP2* variant c.2035C>T in family DCM-23 is recurrent or can be attributed to common ancestry, we performed haplotype analysis using eight repeat markers (including six intragenic *PKP2* markers) within a region of 300,000 base pairs, including the entire genomic region of *PKP2*. The positioning of the gene and the markers are related to the NCBI Build GRCh38.p2. Primers used to amplify these markers were self-designed and are available upon request. The *PKP2* c.2035C>T heterozygous parents, two homozygous siblings, and an unrelated control individual were available for haplotype analysis.

**Whole Exome sequencing**

DNA was isolated from white blood cells using standard techniques (High Pure PCR Template Preparation Kit^®^, Roche Diagnostics GmbH, Mannheim, Germany) and prepared for solution-based hybridization to NimbleGen EZ Exome Library (Roche NimbleGen Inc, Madison, WI, USA) according manufactures instructions (http://sequencing.roche.com). Next generation sequencing was performed on the Illumina HiSeq 2000 sequencing system (Illumina Inc, San Diego, USA) in a 2x100 base paired end mode. For reads alignment and base calling the software tools BWA ([21](#_ENREF_21)), GATK ([22](#_ENREF_22)), and SAMtools ([23](#_ENREF_23)) were used. The detection of variants was restricted to exonic regions and consensus splice sequences (± 1 and 2 position) of those genes listed in the Human Gene Mutation Database (HGMD^®^) Professional 2015.1 ([7](#_ENREF_7)) when using the search term ’cardiomyopathy*’* (for gene list see Tab.S4). As reference the UCSC Genome Build hg19 was used. Variant classification and filter settings were performed as described in the Material and Methods section of the main part of the manuscript.

**Statistics**

Statistical analyses were done using GraphPad PRISM 5.02 software (GraphPad Software Inc.La Jolla, CA). Experimental groups were compared using ANOVA with Bonferroni's post-test or Chi square test, where appropriate. Values of p<0.05 were considered statistically significant.
