# Supplementary material for: High proportion of genetic cases in patients with advanced cardiomyopathy including a novel homozygous Plakophilin 2-gene mutation
Source: PLoS One. 2017 Dec 18;12(12):e0189489. doi: 10.1371/journal.pone.0189489 (PMC5734774; doi:10.1371/journal.pone.0189489)
Supplement: S1 Table — (DOCX) [file pone.0189489.s002.docx]

**S1 Table. Clinical data and family history of DCM and RCM index-patients.**

| **Patient ID** | **Gender** | **Age at diagnosis**  **(Years)** | **Treatment** | **NYHA^1^** | **LVEDD (mm)** | **LV-EF%**  **(FS %)** | **Cardiac Index (CI)** | **Age at HTx^a^/VAD^b^** | **Family history^2^** |
| --- | --- | --- | --- | --- | --- | --- | --- | --- | --- |
| *DCM-01* | m | 1 | VAD, HTx | IV | 67 | 10 | 2.6 | 13**^a^** | De novo |
| *DCM-02* | m | 31 | pre HTx^5^ | III | 70 | 12 | 2.04 | - | De novo |
| *DCM-03* | m | 68 | ICD, VAD | III | 52 | 35 | 2.4 | 69^b^ | Son with DCM |
| *DCM-04* | m | 30 | HTx | IV | 63 | 18 | 2.42 | 62**^a^** | Mother (61y) died from DCM |
| *DCM-05* | m | 11 | HTx | IV | 75 | 32 | 3.1**^4^** | 14 | Older brother with DCM and HTx |
| *DCM-06* | m | < 1 | VAD, HTx | IV | 62 | 15 | n.a. | <1**^a^** | De novo |
| *DCM-07* | m | 35 | ICD, VAD, HTx | IV | 59 | 20 | 2.52 | 47**^a^** | Cousin with HTx, sister with suspicion of DCM, father and brother died from heart disease, sister with heart disease |
| *DCM-08* | m | 23 | VAD, HTx | IV | 68 | 47 | 2,71 | 23^a^ | Father with DCM |
| *DCM-09* | f | 41 | ICD, AMB | II^3,4^ | >55 | 20 | n.a. | - | SCD of father (40y), brother died from heart disease (23y) |
| *DCM-10* | m | 33 | ICD, HTX | IV | 60 | 27 | 1.08 | 37 | Mother and aunt with HTx, brother and niece with DCM |
| *DCM-11* | m | 36 | ICD, HTx | III-IV | 68 | 37 | 2.09 | 48**^a^** | Two cousins died from SCD |
| *DCM-12* | m | 18 | ICD, VAD | III-IV | 91 | 20 | 1.97 | 29**^b^** | De novo |
| *DCM-13* | m | 39 | pre HTx^5^ | I^3,4^ | 65 | 36 | 3.55 | - | Father with DCM and HTx |
| *DCM-14* | m | 60 | ICD, HTx | III | 61 | 25 | 1.7 | 63**^a^** | Mother and two brothers died from heart disease, sister with DCM and HTx |
| *DCM-15* | m | 32 | HTx | III | 83 | 32 | 1,8 | 40**^a^** | Son with DCM and HTx, sister with DCM |
| *DCM-16* | f | 20 | ICD, VAD | III-IV | 75 | 10 | 2.2 | 32**^b^** | De novo. |
| *DCM-17* | m | 14 | ICD,HTx | III | n.a. | 45 | 1.8 | 59 | Father and paternal uncle with SCD |
| *DCM-18* | f | n.a | ICD, HTx | IV | 62 | 20 | 2.63 | 52**^a^** | Monozygotic twin sister (45y) died from heart disease |
| *DCM-19* | m | 5 | ICD, VAD | IV | 74 | 20 | 2.38 | 22**^b^** | De novo |
| *DCM-20* | m | 35 | ICD, pre HTx | II-III | 83 | 14 | 1.8 | - | Father with DCM |
| *DCM-21* | f | n.a | ICD, pre HTx | II^3,4^ | 75 | 28 | 2.81 | - | Father died from DCM, deceased brother with VAD, son and daughter with heart disease |
| *DCM-22* | f | 59 | pre HTx^5^ | II^3,4^ | 65 | 29 | n.a. | - | Brother with HTx, another brother with VAD |
| *DCM-23* | f | n.a. | HTx | IV | 56 | (8%) |  | 14**^a^** | SCD of 2 brothers in childhood, 2 siblings with DCM and HTx in adolescent age |
| *DCM-24* | f | 30 | VAD | IV | 45 | 35 | 2.88 | 31**^b^** | De novo |
| *DCM-25* | m | 40 | ICD, pre HTx | II-III | 87 | 8 | n.a. | - | SCD of 3 maternal uncles, son with DCM |
| *DCM-26* | f | n.a | HTx | IV | 63 | 26 | 2.99^3^ | 16**^a^** | De novo |
| *DCM-27* | m | 38 | VAD, HTx | IV | 72 | 30 | 1.45 | 53**^a^** | Father died from DCM (43y), brother and sister with heart disease |
| *DCM-28* | m | 37 | ICD, VAD, HTx | IV | 72 | 19 | 2.35 | 52**^a^** | SCD of maternal grandmother (60y) |
| *DCM-29* | m | 31 | HTx | IV | 86 | 34 | 2.55 | 42**^a^** | Paternal grandfather (51y) and father (46y) deceased from heart disease |
| *DCM-30* | m | 36 | ICD, pre HTx | II^6^ | 75 | 29 | n.a. | - | De novo |
| *RCM-01* | f | 12 | VAD, HTx | IV | 33 | 51 | 1.9 | 13**^a^** | De novo |
| *RCM-02* | m | < 1 | VAD | IV | normal | 38 | n.a. | <1**^b^** | De novo |
| *RCM-03* | m | 19 | HTx | IV | 47 | 40 | 1.62 | 12**^a^** | Mother (42y) with muscular dystrophy died from heart disease |

**Abbreviations**: **a**=age at HTx, **ACMG class 2**=*likely benign*, **ACMG class 3**=*variant of uncertain significance*, **ACMG class 4**=*likely pathogenic*, **ACMG class 5**=*pathogenic***, AMB**=ambulatory, **b**= age at VAD, **DCM**=dilated cardiomyopathy; **f**=female, **FS**=fractional shortening, **HTx**=heart transplantation, **ICD**=implantable cardioverter defibrillator, **ID**= identification, **LVEDD**=left ventricular end-diastolic diameter in millimeter, **LV-EF**=left ventricular ejection fraction, **m**=male**, n.a.**=not available, **RCM**=restrictive cardiomyopathy, **SCD**=sudden cardiac death, **VAD**=ventricular assist device; **y**=year(s). **^1^**New York Heart Association functional classification of heart failure before VAD or HTx, respectively. ^2^Familial disposition for cardiomyopathy due to pedigree analysis and anecdotal evidence. **^3^**Progressive cardiomyopathy with familial disposition due to pedigree analysis or anecdotal evidence, respectively. ^4^Not yet listed for HTx but in the long term surveillance program. ^5^Treated medically. ^6^Four months before HTx..
