# Supplementary material for: High proportion of genetic cases in patients with advanced cardiomyopathy including a novel homozygous Plakophilin 2-gene mutation
Source: PLoS One. 2017 Dec 18;12(12):e0189489. doi: 10.1371/journal.pone.0189489 (PMC5734774; doi:10.1371/journal.pone.0189489)
Supplement: S2 Table — (DOCX) [file pone.0189489.s003.docx]

**S2 Table. Task force classification and family history of ARVC index-patients.**

| **Patient ID** | **Gender** | **Age at diagnosis (Years)** | **Treatment** | **Major criteria^1^** | **Minor criteria^1^** | **Task force classification^2^** | **Age at HTx^a^/VAD^b^** | **Family history^3^** |
| --- | --- | --- | --- | --- | --- | --- | --- | --- |
| *ARVC-01* | m | 14 | ICD | (1) RVEDD 42 mm and dyskinesia, (2) pathogenic mutation |  | DD |  | No |
| *ARVC-02* | m | 35 | ICD, HTx | (1) RVEDD 61 mm and dyskinesia, (2) ARVC confirmed by examination of the explanted heart |  | DD | 36**^a^** | SCD of father, brother with heart disease |
| *ARVC-03* | m | 67 | ICD |  | (1) RVEDD 35mm with normal LV, (2) > 500 ventricular extrasystoles per 24 h | PD |  | No |
| *ARVC-04* | f | 35 | ICD, TAH | (1) RVEDD 44 mm and dyskinesia, (2) pathogenic mutation |  | DD | 55**^b^** | SCD of maternal grandmother and uncle, sister with DCM |
| *ARVC-05* | f | 45 | ICD, HTx | (1) RVEDD 44mm and dyskinesia, (2) pathogenic mutation |  | DD | 47**^a^** | Maternal grandfather with SCD |
| *ARVC-06* | f | 21 | ICD, HTx | (1) RVEDD >62mm and dyskinesia, (2) residual myocytes <50% with fibrous replacement of the RV of the explanted heart, (3) pathogenic mutation |  | DD | 32**^a^** | ARVC with SCD of father and brother |
| *ARVC-07* | m | 40 | ICD, HTx | (1) RVEDD >48mm and dyskinesia | (1) 50%-65% residual myocytes with fibrous replacement of the RV of the explanted heart | BL | 62**^a^** | Paternal great-grandfather and father with heart disease and premature death |
| *ARVC-08* | m | 16 | ICD, HTx | (1) RVEDD >67mm and dyskinesia | (1) >500 ventricular extrasystoles per 24h | BL | 21**^a^** | No |
| *ARVC-09* | f | 42 | VAD | (1) RVEDD >40mm and dyskinesia |  | PD | 45**^b^** | No |
| *ARVC-10* | f | n.a | ICD, HTx | (1) RVEDD >45mm and dyskinesia, (2) residual myocytes <50% with fibrous replacement of the RV of the explanted heart |  | DD | 46**^a^** | Two maternal great-uncles with SCD |

**Abbreviations**: **a**=age at HTx, **ARVC**=arrhythmogenic right ventricular cardiomyopathy, **b**=age at VAD, **BSA**=body surface area**, f**=female, **h**=hour(s), **HTx**=heart transplantation, **ICD=** implantable cardioverter defibrillator**,** **LV**=left ventricle, **m**=male, **MRI**=magnetic resonance imaging; **n.a.**= not available, **RV**=right ventricle, **RVEDD**=right ventricular end-diastolic diameter in millimeter, **TAH**=total artificial heart; **VT**=ventricular tachycardia; **y**=year(s). **^1^**Task force criteria according to ref.([24](#_ENREF_24)) and adapted criteria for RVEDD data: major criterion, RVEDD ≥ 40mm and RV akinesia, dyskinesia, or aneurysm; minor criterion, RVEDD ≤ 40mm to ≥ 35mm and RV akinesia, dyskinesia, or aneurysm. **^2^**Disease classification according to Task Force Criteria ([24](#_ENREF_24)); **BL**=borderline diagnosis, **DD**=definite diagnosis; **PD**=possible diagnosis of ARVC. **^3^**Familial disposition for cardiomyopathy due to pedigree analysis and anecdotal evidence.
