# Supplementary material for: High proportion of genetic cases in patients with advanced cardiomyopathy including a novel homozygous Plakophilin 2-gene mutation
Source: PLoS One. 2017 Dec 18;12(12):e0189489. doi: 10.1371/journal.pone.0189489 (PMC5734774; doi:10.1371/journal.pone.0189489)
Supplement: S3 Table — (DOCX) [file pone.0189489.s004.docx]

**S3 Table.** Illumina myopathy panel 46 gene list.

Reference sequences are given by the National Center of Biotechnology Information (NCBI, www.ncbi.nlm.nih.gov). Phenotype MIM numbers refers to the informations given at <http://omim.org>.

|  |  |  |  |  |
| --- | --- | --- | --- | --- |
| **Gene symbol** | **Reference sequence** | **Reference protein** | **Disease** | **Phenotype MIM number** |
| ABCC9 | NM_005691.2 | NP_005682.2 | CMD10 | 608569 |
| ACTC1 | NM_005159.4 | NP_005150.1 | CMD1R, LVNC4 | 613424 |
| ACTN2 | NM_001103.3 | NP_001094.1 | CMD1AA, CMH23 | 612158 |
| ANKRD1 | NM_014391.2 | NP_055206.2 | CMD1KK, CMH22, RCM4 | 615248 |
| CASQ2 | NM_001232 | NP_001223 | CPVT | 611938 |
| CAV3 | NM_033337.2 | NP_203123.1 | CMH | 192600 |
| CRYAB | NM_001885.2 | NP_001876 | CMD1II | 615184 |
| CSRP3 | NM_003476.4 | NP_003467.1 | CMD1M, CMH12 | 607482, 612124 |
| CTF1 | NM_001330.3 | NP_001321.1 | CMD | n.a. |
| DES | NM_001927.3 | NP_001918.3 | CMD1I | 604765 |
| DSC2 | NM_024422.3 | NP_077740.1 | ARVD11 | 610476 |
| DSG2 | NM_001943.3 | NP_001934.2 | ARVD10, CMD1BB | 610193, 612877 |
| DSP | NM_004415.2 | NP_004406.2 | ARVD8,CMD | 607450, 605676 |
| DTNA | NM_001390.4 | NP_001381.2 | LVNC1 | 604169 |
| EMD | NM_000117.2 | NP_000108.1 | EMD, CMD | 310300 |
| FHL2 | NM_201555.1 | NP_963849.1 | CMD | n.a. |
| GLA | NM_000169.2 | NP_000160.1 | Fabry disease, CMH | 301500 |
| JUP | NM_021991.2 | NP_068831.1 | ARVD12 | 611528 |
| LAMA4 | NM_001105206.2 | NP_001098676.2 | CMD1JJ | 615235 |
| LAMP2 | NM_001122606.1 | NP_001116078.1 | Danon disease, CMD | 300257 |
| LDB3 | NM_001171610.1 | NP_001165081.1 | CMD1C, CMH24, LVNC3 | 601493 |
| LMNA | NM_170707.2 | NP_733821.1 | CMD1A | 115200 |
| MYH6 | NM_002471.3 | NP_002462.2 | CMD1EE, CMH14 | 613252, 613251 |
| MYH7 | NM_000257.2 | NP_000248.2 | CMD1S, CMH1, LVNC5 | 613426, 192600 |
| MYL2 | NM_000432.3 | NP_000423.2 | CMH10 | 608758 |
| MYL3 | NM_000258.2 | NP_000249.1 | CMH8 | 608751 |
| MYLK2 | NM_033118.3 | NP_149109.1 | CMH1 | 192600 |
| MYOZ2 | NM_016599.4 | NP_057683.1 | CMH16 | 613838 |
| NEXN | NM_144573.3 | NP_653174.3 | CMD1CC, CMH20 | 613122, 613876 |
| PKP2 | NM_004572.3 | NP_004563.2 | ARVD9 | 609040 |
| PLN | NM_002667.4 | NP_002658 | CMD1P, CMH18 | 609909, 613874 |
| PRKAG2 | NM_016203.3 | NP_057287.2 | CMH6 | 600858 |
| RBM20 | NM_001134363.1 | NP_001127835.1 | CMD1DD | 613172 |
| RYR2 | NM_001035.2 | NP_001026.2 | ARVD2 | 600996 |
| SGCD | NM_000337.5 | NP_000328.2 | CMD1L | 606685 |
| TAZ | NM_000116.3 | NP_000107.1 | Barth syndrome, CMD, LVNC | 302060 |
| TCAP | NM_003673.3 | NP_003664 | CMH25 | 607487 |
| TMEM43 | NM_024334.2 | NP_077310.1 | ARVD5 | 604400 |
| TNNC1 | NM_003280.2 | NP_003271 | CMD1Z, CMH13 | 611879, 613242 |
| TNNI3 | NM_000363.4 | NP_000354.4 | CMD2A, CMD1FF, CMR, CMH | 611880, 613286, 115210, 613690 |
| TNNT2 | NM_001001430.2 | NP_001001430.1 | CMD1D, CMR, CMH, LVNC | 601494, 612422, 115195, 601494 |
| TPM1 | NM_001018005.1 | NP_001018005.1 | CMD1Y, CMH3, LVNC | 611878, 115196, 611878 |
| TTN | NM_001267550.1 | NP_001254479.1 | CMD1G, CMH | 604145, 613765 |
| TTR | NM_000371.3 | NP_000362.1 | Cardiomyopathy | n.a. |
| VCL | NM_014000.2 | NP_054706.1 | CMD1W, CMH15 | 611407, 613255 |

Abbreviation: n.a.=Not available
