# Supplementary material for: High proportion of genetic cases in patients with advanced cardiomyopathy including a novel homozygous Plakophilin 2-gene mutation
Source: PLoS One. 2017 Dec 18;12(12):e0189489. doi: 10.1371/journal.pone.0189489 (PMC5734774; doi:10.1371/journal.pone.0189489)
Supplement: S4 Table — (DOCX) [file pone.0189489.s005.docx]

**S4 Table**. 46-gene panel next generation sequencing quality data with specification of read depth and target coverage.

| Index patient ID | mean region coverage depth | target coverage at 20x (%) | target coverage at 50x |
| --- | --- | --- | --- |
| DCM-01 | 693 | 99,8 | 99,6 |
| DCM-02 | 787 | 100 | 99,7 |
| DCM-03 | 633 | 99,9 | 99,8 |
| DCM-04 | 284 | 93,9 | 91 |
| DCM-05 | 865 | 96,6 | 94,8 |
| DCM-06 | 487 | 99,9 | 99,6 |
| DCM-07 | 671 | 100 | 99,8 |
| DCM-08 | 725 | 99,9 | 99,2 |
| DCM-09 | 959 | 100 | 99,8 |
| DCM-10 | 340 | 99,4 | 97,8 |
| DCM-11 | 929 | 100 | 99,8 |
| DCM-12 | 871 | 100 | 100 |
| DCM-13 | 774 | 100 | 99,7 |
| DCM-14 | 983 | 100 | 99,9 |
| DCM-15 | 846 | 100 | 99,9 |
| DCM-16 | 760 | 100 | 99,9 |
| DCM-17 | 662 | 99,8 | 99,2 |
| DCM-18 | 635 | 99,8 | 99,1 |
| DCM-19 | 849 | 100 | 100 |
| DCM-20 | 490 | 99,9 | 98,8 |
| DCM-21 | 970 | 100 | 99,8 |
| DCM-22 | 594 | 99,9 | 99,4 |
| DCM-23 | 468 | 99,9 | 99,6 |
| DCM-24 | 1217 | 100 | 99,9 |
| DCM-25 | 661 | 99,9 | 99,8 |
| DCM-26 | 620 | 99,9 | 99,7 |
| DCM-27 | 794 | 100 | 99,8 |
| DCM-28 | 698 | 99,9 | 99,5 |
| DCM-29 | 667 | 99,9 | 99,4 |
| DCM-30 | 641 | 99,9 | 99,5 |
| LVNC-01 | 796 | 100 | 99,6 |
| HNOCM-01 | 1472 | 100 | 100 |
| RCM-01 | 624 | 97,3 | 95,8 |
| RCM-02 | 537 | 99,8 | 99,5 |
| RCM-03 | 398 | 98,6 | 95,3 |
| ARVC-01 | 734 | 100 | 99,9 |
| ARVC-02 | 1228 | 100 | 99,9 |
| ARVC-03 | 1167 | 100 | 99,9 |
| ARVC-04 | 947 | 100 | 99,9 |
| ARVC-05 | 906 | 99,8 | 99,5 |
| ARVC-06 | 887 | 100 | 99,9 |
| ARVC-07 | 699 | 99,8 | 99,2 |
| ARVC-08 | 703 | 99,9 | 99,4 |
| ARVC-09 | 1020 | 100 | 99,9 |
| ARCV-10 | 843 | 99,9 | 99,5 |
| ARVC-11 | 772 | 99,9 | 99,3 |
| ARVC-12 | 898 | 99,9 | 99,7 |
| ARVC-13 | 958 | 100 | 99,8 |
| ARVC-14 | 575 | 99,9 | 99,5 |
| ARVC-15 | 1059 | 100 | 99,9 |
| Mean | 761,6037736 | 99,66226415 | 99,13018868 |
