# Supplementary material for: High proportion of genetic cases in patients with advanced cardiomyopathy including a novel homozygous Plakophilin 2-gene mutation
Source: PLoS One. 2017 Dec 18;12(12):e0189489. doi: 10.1371/journal.pone.0189489 (PMC5734774; doi:10.1371/journal.pone.0189489)
Supplement: S5 Table — (DOCX) [file pone.0189489.s006.docx]

**S5 Table. Overview on genotyping results and variation classification according to the ACMG guidelines.**

|  | |  | | | | | | |  |  |
| --- | --- | --- | --- | --- | --- | --- | --- | --- | --- | --- |
| **Patient** | **Affected gene** | | **Nucleotide**  **change^1^** | **Amino acid change^1^** | **Haplotype** | **rs number^2^** | **Co-segregation** | **Pathogenic evidence according ACMG^3^** | | **Ref.** |
| DCM-01 | *MYL2* | | c.401A>C | p.Glu134Ala | het | rs143139258 | Unknown^7^ | 4 | | [[1](#_ENREF_1), [2](#_ENREF_2)] |
|  | *MYH7* | | c.5390T>C | p.Leu1797Pro | het |  | De novo | 4 | |  |
| DCM-02 | *TTN* | | c.25570G>A | p.Gly8524Arg^4^ | het | rs371512914 | n.t. | 3 | |  |
| DCM-03 | *TTN* | | c.65035_65036 delGC | p.Ala21679fLeufs*6 | het |  | Yes | 4 | |  |
|  | *DSC2* | | c.1307G>T | p.Gly436Val | het |  | No | 2 | |  |
|  | *TTN* | | c.92595A>C | p.Leu30865Phe | het |  | Yes | 3 | |  |
| DCM-04 | *TTN* | | c.61682C>G | p.Ser20561* | het |  | n.t. | 4 | |  |
|  | *DSP* | | c.1430A>G | p.His477Arg | het |  | n.t. | 3 | |  |
| DCM-05 | *TNNC1* | | c.435C>A | p.Asp145Glu | het | rs267607124 | Yes | 4 | | [[3-6](#_ENREF_3)] |
|  | *TNNC1* | | c.184G>A | p.Asp62Asn | het |  | Yes | 3 | |  |
| DCM-06 | *TTN* | | c.55745C>T | p.Pro18582Leu | het | rs201194435 | n.t. | 3 | |  |
| DCM-07 | *LMNA* | | c.568C>T | p.Arg190Trp | het | rs59026483 | n.t. | 5 | | [[7-9](#_ENREF_7)] |
| DCM-08 | *TTN* | | c.41486G>C | p.Gly13829Ala | het |  | n,t, | 3 | |  |
| DCM-09 | *DES* | | c.1332_1335 delGACG | p.Thr445* | het |  | n.t. | 5 | |  |
|  | *TTN* | | c.86637T>A | p.Asn28879Lys | het |  | n.t. | 3 | |  |
|  | *TTN* | | c.83062C>T | p.Arg27688Cys | het |  | n.t. | 3 | |  |
| DCM-10 | *DSP* | | c.3551G>A | p.Arg1184Gln | het | rs147909031 | No | 2 | |  |
| DCM-11 | *DES* | | c.407T>C | p.Leu136Pro | het |  | Unknown^7^ | 4 | | [[10](#_ENREF_10)] |
| DCM-12 | *TNNT2* | | c.644G>A | p.Arg215Lys | het |  | n.t. | 3 | |  |
|  | *TTN* | | c.12438_12448 del11 | p.Ser4147Thrfs*20**^5^** | het |  | n.t. | 4 | |  |
| DCM-13 | *LMNA* | | c.1634G>A | p.Arg545His | het | rs142191737 | n.t. | 4 | | [[11](#_ENREF_11)] |
|  | *TTN* | | c.87355delG | p.Ala29119Leufs*17 | het |  | n.t. | 4 | |  |
| DCM-14 | *RBM20* | | c.1904C>G | p.Ser635Cys | het |  | Unknown^7^ | 4 | |  |
|  | *DSP* | | c.3616T>A | p.Leu1206Ile | het | rs151115778 | Unknown^7^ | 3 | |  |
| DCM-15 | *RBM20* | | c.1913C>T | p.Pro638Leu | het | rs267697003 | Yes | 5 | | [[12](#_ENREF_12)] |
|  | *TTN* | | c.54140C>T | p.Ala18047Val | het | rs373815064 | No | 3 | |  |
| DCM-16 | *TTN* | | c.51436+1G>A |  | het |  | n.t. | 4 | |  |
|  | *TTN* | | c.11887G>A | p.Gly3963Arg**^5^** | het |  | n.t. | 3 | |  |
| DCM-17 | *DES* | | c.493_520 delinsGCGT | p.Gln165_Ala174 delinsAlaSer | het |  | Unknown^8^ | 5 | |  |
| DCM-18 | *LMNA* | | c.908_909delCT | p.Ser303Cysfs*27 | het | rs59684335 | n.t. | 5 | | [[13](#_ENREF_13), [14](#_ENREF_14)] |
| DCM-19 | *NEXN* | | c.1955A>G | p.Tyr652Cys | het | rs137853197 | n.t. | 4 | | [[15](#_ENREF_15)] |
|  | *MYH7* | | c.1106G>A | p.Arg369Gln | het | rs397516089 | n.t. | 4 | | [[16](#_ENREF_16)] |
| DCM-20 | *TTN* | | c.42909_42910 delTG | p.Cys14303Trpfs*12 | het |  | n.t. | 4 | |  |
|  | *DSP* | | c.136G>A | p.Gly46Ser | het | rs371517189 | n.t. | 3 | |  |
| DCM-21 | *TTN* | | c.521A>G | p.Tyr174Cys | het |  | n.t. | 3 | |  |
| DCM-22 | *TTN* | | c.54768delT | p.Ser18258Valfs*34 | het |  | n.t. | 4 | |  |
| DCM-23 | *PKP2* | | c.2035C>T | p.His679Tyr | hom |  | Yes | 4 | |  |
|  | *LAMA4* | | c.133C>T | p.Gln45* | het |  | No | 2 | |  |
| DCM-24 | *TTN* | | c.101774_101776 dupAAG | p.Glu33925dup | het |  | n.t. | 3 | |  |
|  | *TTN* | | c.106403T>A | p.Leu35468His | het |  | n.t. | 3 | |  |
|  | *TTN* | | c.74305A>G | p.Asn24769Asp | het | rs372787601 | n.t. | 3 | |  |
| RCM-01 | *MYL3* | | c.461G>A | p.Arg154His | hom | rs104893749 | Unkown^8^ | 3**^6^** | | [[17](#_ENREF_17), [18](#_ENREF_18)] |
| RCM-02 | *TNNI3* | | c.379G>T | p. Asp127Tyr | het |  | De novo | 4 | | [[19](#_ENREF_19)] |
| RCM-03 | *CRYAB* | | c.326A>G | p.Asp109Gly | het |  | n.t. | 5 | | [[20](#_ENREF_20)] |
| ARVC-01 | *PKP2* | | c.2146-1G>C |  | het | rs193922674 | n.t. | 5 | | [[21-23](#_ENREF_21)] |
| ARVC-02 | *MYH7* | | c.3715A>G | p.Ile1239Val | het |  | n.t. | 3 | |  |
| ARVC-03 | *PRKAG2* | | c.425C>T | p.Thr142Ile | het | rs397517270 | n.t. | 3 | |  |
| ARVC-04 | *PLN* | | c.40_42delAGA | p.Arg14del | het |  | Yes | 5 | | [[24-26](#_ENREF_24)] |
|  | *MYH6* | | c.3607dupG | p.Ala1203Glyfs*30 | het |  | Yes | 3 | |  |
|  | *TTN* | | c.59113C>T | p.Arg19705Cys | het |  | No | 2 | |  |
| ARVC-05 | *LMNA* | | c.1073A>G | p.Glu358Gly | het |  | n.t. | 4 | |  |
|  | *PKP2* | | c.2326T>C | p.Ser776Pro | het |  | n.t. | 3 | |  |
| ARVC-06 | *PKP2* | | c.2146-1G>C |  | het | rs193922674 | n.t. | 5 | | [[21-23](#_ENREF_21)] |
|  | *PKP2* | | c.1138G>A | p.Glu380Lys | het |  | n.t. | 3 | |  |
|  | *RYR2* | | c.4069G>A | p.Asp1357Asn | het |  | n.t. | 3 | |  |
|  | *TTN* | | c.102877A>G | p.Lys34293Glu | het | rs72629783 | n.t. | 3 | |  |
| ARVC-07 | *DES* | | c.1315G>A | p.Glu439Lys | het |  | n.t. | 3 | | [[27](#_ENREF_27)] |

**Abbreviations**: **ARVC**=arrhythmogenic right ventricular cardiomyopathy, **DCM**=dilated cardiomyopathy, **het**=heterozygous, **hom**=homozygous; n.t.=not tested, **RCM**=restrictive cardiomyopathy. **^1^**For reference sequence number see S1 Table, for nomenclature of sequence variants see Material and Methods section. **^2^**Nomenclature according to dbSNP (www.ncbi.nlm.nih.gov/snp/). **^3^**Pathogenic evidence according to the ACMG guidelines [[28](#_ENREF_28)]. **^4^**Affects only *TTN*-isoform N2BA. **^5^**Affects only *TTN*-isoform N2B. Unmarked *TTN*-variation affects TTN-N2BA and TTN-N2B. **^6^**See S2 Fig. Family histories and variant co-segregation. ^7^Healthy family member is carrier of the variant too. ^8^Too few family members available.

1. Olivotto, I., et al., *Myofilament protein gene mutation screening and outcome of patients with hypertrophic cardiomyopathy.* Mayo Clin Proc, 2008. **83**(6): p. 630-8.

2. Burghardt, T.P. and L.A. Sikkink, *Regulatory light chain mutants linked to heart disease modify the cardiac myosin lever arm.* Biochemistry, 2013. **52**(7): p. 1249-59.

3. Pinto, J.R., et al., *A functional and structural study of troponin C mutations related to hypertrophic cardiomyopathy.* J Biol Chem, 2009. **284**(28): p. 19090-100.

4. Swindle, N. and S.B. Tikunova, *Hypertrophic cardiomyopathy-linked mutation D145E drastically alters calcium binding by the C-domain of cardiac troponin C.* Biochemistry, 2010. **49**(23): p. 4813-20.

5. Pinto, J.R., et al., *Strong cross-bridges potentiate the Ca(2+) affinity changes produced by hypertrophic cardiomyopathy cardiac troponin C mutants in myofilaments: a fast kinetic approach.* J Biol Chem, 2011. **286**(2): p. 1005-13.

6. Pinto, J.R., et al., *Functional characterization of TNNC1 rare variants identified in dilated cardiomyopathy.* J Biol Chem, 2011. **286**(39): p. 34404-12.

7. Bhattacharjee, P., et al., *Structural alterations of Lamin A protein in dilated cardiomyopathy.* Biochemistry, 2013. **52**(24): p. 4229-41.

8. Quarta, G., et al., *Mutations in the Lamin A/C gene mimic arrhythmogenic right ventricular cardiomyopathy.* Eur Heart J, 2012. **33**(9): p. 1128-36.

9. Sylvius, N., et al., *In vivo and in vitro examination of the functional significances of novel lamin gene mutations in heart failure patients.* J Med Genet, 2005. **42**(8): p. 639-47.

10. Brodehl, A., et al., *Functional characterization of the novel DES mutation p.L136P associated with dilated cardiomyopathy reveals a dominant filament assembly defect.* J Mol Cell Cardiol, 2015. **91**: p. 207-214.

11. van Rijsingen, I.A., et al., *Gender-specific differences in major cardiac events and mortality in lamin A/C mutation carriers.* Eur J Heart Fail, 2013. **15**(4): p. 376-84.

12. Guo, W., et al., *RBM20, a gene for hereditary cardiomyopathy, regulates titin splicing.* Nat Med, 2012. **18**(5): p. 766-73.

13. MacLeod, H.M., et al., *Lamin A/C truncation in dilated cardiomyopathy with conduction disease.* BMC Med Genet, 2003. **4**: p. 4.

14. Sparks, E.A., et al., *Heritable Cardiac Conduction and Myocardial Disease: From the Clinic to the Basic Science Laboratory and Back to the Clinic.* Cardiology, 2011. **118**(3): p. 179-186.

15. Hassel, D., et al., *Nexilin mutations destabilize cardiac Z-disks and lead to dilated cardiomyopathy.* Nat Med, 2009. **15**(11): p. 1281-8.

16. Dellefave, L.M., et al., *Sarcomere mutations in cardiomyopathy with left ventricular hypertrabeculation.* Circ Cardiovasc Genet, 2009. **2**(5): p. 442-9.

17. Poetter, K., et al., *Mutations in either the essential or regulatory light chains of myosin are associated with a rare myopathy in human heart and skeletal muscle.* Nat Genet, 1996. **13**(1): p. 63-9.

18. Lossie, J., et al., *Mutations of ventricular essential myosin light chain disturb myosin binding and sarcomeric sorting.* Cardiovasc Res, 2012. **93**(3): p. 390-6.

19. Sheng, H.Z., et al., *[Cardiac troponin I gene mutation (Asp127Tyr) in a Chinese patient with hypertrophic cardiomyopathy].* Zhonghua Xin Xue Guan Bing Za Zhi, 2008. **36**(12): p. 1063-5.

20. Brodehl, A., et al., *The novel alphaB-crystallin (CRYAB) mutation p.D109G causes restrictive cardiomyopathy.* Hum Mutat, 2017. **38**(8): p. 947-952.

21. Gerull, B., et al., *Mutations in the desmosomal protein plakophilin-2 are common in arrhythmogenic right ventricular cardiomyopathy.* Nat Genet, 2004. **36**(11): p. 1162-4.

22. Cox, M.G., et al., *Arrhythmogenic right ventricular dysplasia/cardiomyopathy: pathogenic desmosome mutations in index-patients predict outcome of family screening: Dutch arrhythmogenic right ventricular dysplasia/cardiomyopathy genotype-phenotype follow-up study.* Circulation, 2011. **123**(23): p. 2690-700.

23. Fressart, V., et al., *Desmosomal gene analysis in arrhythmogenic right ventricular dysplasia/cardiomyopathy: spectrum of mutations and clinical impact in practice.* Europace, 2010. **12**(6): p. 861-8.

24. Haghighi, K., et al., *A mutation in the human phospholamban gene, deleting arginine 14, results in lethal, hereditary cardiomyopathy.* Proc Natl Acad Sci U S A, 2006. **103**(5): p. 1388-93.

25. Karakikes, I., et al., *Correction of human phospholamban R14del mutation associated with cardiomyopathy using targeted nucleases and combination therapy.* Nat Commun, 2015. **6**: p. 6955.

26. Posch, M.G., et al., *Genetic deletion of arginine 14 in phospholamban causes dilated cardiomyopathy with attenuated electrocardiographic R amplitudes.* Heart Rhythm, 2009. **6**(4): p. 480-6.

27. Wahbi, K., et al., *High cardiovascular morbidity and mortality in myofibrillar myopathies due to DES gene mutations: a 10-year longitudinal study.* Neuromuscul Disord, 2012. **22**(3): p. 211-8.

28. Richards, S., et al., *Standards and guidelines for the interpretation of sequence variants: a joint consensus recommendation of the American College of Medical Genetics and Genomics and the Association for Molecular Pathology.* Genet Med, 2015. **17**(5): p. 405-24.
