# Supplementary material for: High proportion of genetic cases in patients with advanced cardiomyopathy including a novel homozygous Plakophilin 2-gene mutation
Source: PLoS One. 2017 Dec 18;12(12):e0189489. doi: 10.1371/journal.pone.0189489 (PMC5734774; doi:10.1371/journal.pone.0189489)
Supplement: S6 Table — (DOCX) [file pone.0189489.s007.docx]

**S6 Table. Considered criteria for variant classification according to the ACMG guidelines.**

|  |  |  |  |  | **ACMG criteria^2^** | | | | | | | | | | | | | | |  |  |
| --- | --- | --- | --- | --- | --- | --- | --- | --- | --- | --- | --- | --- | --- | --- | --- | --- | --- | --- | --- | --- | --- |
| **Patient** | **Affected gene** | **Nucleotide change^1^** | **Amino acid change**^1^ | **PVS1** | | **PS1** | **PS3** | **PM2^*^** | **PM4** | **PM5** | **PM6** | **PP2** | **PP3** | **PP4** | **PP5** | **BS2** | **BS4** | **BP4** | **BP5** | **ACMG class**^2^ | **Reference**^3^ |
|  |  |  |  |  |  |  |  |  |  |  |  |  |  |  |  |  |  |  |  |  |  |
| *DCM-01* | MYL2 | c.401A>C | p.Glu134Ala |  | |  | y | y |  |  |  |  | y | y | y |  |  |  | y | 4 |  |
|  | MYH7 | c.5390T>C | p.Leu1797Pro |  | |  |  | y |  |  | y | y | y | y |  |  |  |  | y | 4 |  |
| *DCM-02* | TTN | c.25570G>A | p.Gly8524Arg^4^ |  | |  |  | y |  |  |  |  |  | y |  |  |  |  |  | 3 |  |
| *DCM-03* | TTN | c.65035_65036 delGC | p.Ala21679Leufs*6 | Y | |  |  | y |  |  |  |  |  | y |  |  |  |  |  | 4 | ([18](#_ENREF_18), [19](#_ENREF_19)) |
|  | DSC2 | c.1307G>T | p.Gly436Val |  | |  |  | y |  |  |  |  |  | y |  |  | y | y | y | 2 |  |
|  | TTN | c.92595A>C | p.Leu30865Phe |  | |  |  | y |  |  |  |  |  | y |  |  |  |  | y | 3 |  |
| *DCM-04* | TTN | c.61682C>G | p.Ser20561* | Y | |  |  | y |  |  |  |  |  | y |  |  |  |  |  | 4 | ([18](#_ENREF_18), [19](#_ENREF_19)) |
|  | DSP | c.1430A>G | p.His477Arg |  | |  |  | y |  |  |  |  |  | y |  |  |  | Y | y | 3 |  |
| *DCM-05* | TNNC1 | c.435C>A | p.Asp145Glu |  | |  | y | y |  |  |  |  | y | y | y |  |  |  |  | 4 | ([27-30](#_ENREF_27)) |
|  | TNNC1 | c.184G>A | p.Asp62Asn |  | |  |  | y |  |  |  |  |  | y |  |  |  | y | y | 3 |  |
| *DCM-06* | TTN | c.55745C>T | p.Pro18582Leu |  | |  |  | y |  |  |  |  |  | y |  |  |  | y |  | 3 |  |
| *DCM-07* | LMNA | c.568C>T | p.Arg190Trp |  | | y | y | y |  |  |  |  | y | y |  |  |  |  |  | 5 |  |
| *DCM-08* | TTN | c.41486G>C | p.Gly13829Ala |  | |  |  | y |  |  |  |  |  | y |  |  |  | y |  | 3 |  |
| *DCM-09* | DES | c.1332_1335 delGACG | p.Thr445* | Y | |  |  | y |  |  |  |  |  | y |  |  |  |  |  | 5 | ([52](#_ENREF_52), [53](#_ENREF_53)) |
|  | TTN | c.86637T>A | p.Asn28879Lys |  | |  |  | y |  |  |  |  |  | y |  |  |  |  | y | 3 |  |
|  | TTN | c.83062C>T | p.Arg27688Cys |  | |  |  | y |  |  |  |  |  | y |  |  |  |  | y | 3 |  |
| *DCM-10* | DSP | c.3551G>A | p.Arg1184Gln |  | |  |  | y |  |  |  |  |  | y |  |  | y |  |  | 2 |  |
| *DCM-11* | DES | c.407T>C | p.Leu136Pro |  | |  | y | y |  |  |  |  | y | y |  |  |  |  |  | 4 |  |
| *DCM-12* | TNNT2 | c.644G>A | p.Arg215Lys |  | |  |  | y |  |  |  |  | y | y |  |  |  |  | y | 3 |  |
|  | TTN | c.12438_12448 del11 | p.Ser4147Thrfs*20^5^ | Y | |  |  | y |  |  |  |  |  | y |  |  |  |  |  | 4 | ([18](#_ENREF_18), [19](#_ENREF_19)) |
| *DCM-13* | LMNA | c.1634G>A | p.Arg545His |  | |  |  | y |  | y |  |  |  | y | y |  |  |  | y | 4 | ([54](#_ENREF_54)) |
|  | TTN | c.87355delG | p.Ala29119Leufs*17 | Y | |  |  | y |  |  |  |  |  | y |  |  |  |  | y | 4 | ([18](#_ENREF_18), [19](#_ENREF_19)) |
| *DCM-14* | RBM20 | c.1904C>G | p.Ser635Cys |  | |  | y**^6^** | y |  | Y |  |  |  | y |  |  |  |  |  | 4 | ([36](#_ENREF_36)) |
|  | DSP | c.3616T>A | p.Leu1206Ile |  | |  |  | y |  |  |  |  |  | y |  |  |  | y | y | 3 |  |
| *DCM-15* | RBM20 | c.1913C>T | p.Pro638Leu |  | | y | y | y |  |  |  |  |  | y |  |  |  |  |  | 5 |  |
|  | TTN | c.54140C>T | p.Ala18047Val |  | |  |  | y |  |  |  |  |  | y |  |  | y |  | y | 3 |  |
| *DCM-16* | TTN | c.51436+1G>A |  | Y | |  |  | y |  |  |  |  |  | y |  |  |  |  |  | 4 | ([18](#_ENREF_18), [19](#_ENREF_19)) |
|  | TTN | c.11887G>A | p.Gly3963Arg**^5^** |  | |  |  | y |  |  |  |  |  | y |  |  |  |  | y | 3 |  |
| *DCM-17* | DES | c.493_520 delinsGCGT | p.Gln165_Ala174 delinsAlaSer | Y | |  |  | y |  |  |  |  |  | y |  |  |  |  |  | 5 | ([52](#_ENREF_52), [53](#_ENREF_53)) |
| *DCM-18* | LMNA | c.908_909delCT | p.Ser303Cysfs*27 | Y | | y |  | y |  |  |  |  |  | y |  |  |  |  |  | 5 | ([35](#_ENREF_35)) |
| *DCM-19* | NEXN | c.1955A>G | p.Tyr652Cys |  | |  | y | y |  |  |  |  | y | y | y |  |  |  | y | 4 |  |
|  | MYH7 | c.1106G>A | p.Arg369Gln |  | |  |  | y |  |  |  | y | y | y | y |  |  |  | y | 4 |  |
| *DCM-20* | TTN | c.42909_42910 delTG | p.Cys14303Trp*12fs | Y | |  |  | y |  |  |  |  |  | y |  |  |  |  |  | 4 | ([18](#_ENREF_18), [19](#_ENREF_19)) |
|  | DSP | c.136G>A | p.Gly46Ser |  | |  |  | y |  |  |  |  |  | y |  |  |  | y | y | 3 |  |
| *DCM-21* | TTN | c.521A>G | p.Tyr174Cys |  | |  |  | y |  |  |  |  | y | y |  |  | y |  |  | 3 |  |
| *DCM-22* | TTN | c.54768 | p.Ser18258Valfs*34 | Y | |  |  | y |  |  |  |  |  | y |  |  |  |  |  | 4 | ([18](#_ENREF_18), [19](#_ENREF_19)) |
| *DCM-23* | PKP2 | c.2035C>T | p.His679Tyr |  | |  |  | y | y**^7^** |  |  |  | y | y |  | y**^8^** |  |  |  | 4 |  |
|  | LAMA4 | c.133C>T | p.Gln45* |  | |  |  | y |  |  |  |  |  | y |  | y | y |  | y | 2 |  |
| *DCM-24* | TTN | c.101774_101776 dupAAG | p.Glu33925dup |  | |  |  | y | y |  |  |  |  | y |  |  |  |  |  | 3 |  |
|  | TTN | c.106403T>A | p.Leu35468His |  | |  |  | y |  |  |  |  |  | y |  |  |  |  |  | 3 |  |
|  | TTN | c.74305A>G | p.Asn24769Asp |  | |  |  | y |  |  |  |  |  | y |  |  |  | y |  | 3 |  |
| *RCM-01* | MYL3 | c.461G>A | p.Arg154His |  | |  | y | y |  |  |  |  | y | y | y |  |  |  |  | 3**^6^** |  |
| *RCM-02* | TNNI3 | c.379G>T | p. Asp127Tyr |  | |  |  | y |  |  | y |  | y | y | y |  |  |  |  | 4 |  |
| *RCM-03* | CRYAB | c.326A>G | p.Asp109Gly |  | |  | y | y |  | y |  |  | y | y |  |  |  |  |  | 5 | ([55](#_ENREF_55), [56](#_ENREF_56)) |
| *ARVC-01* | PKP2 | c.2146-1G>C |  | Y | |  | y | y |  |  |  |  |  | y | y |  |  |  |  | 5 |  |
| *ARVC-02* | MYH7 | c.3715A>G | p.Ile1239Val |  | |  |  | y |  |  |  | y |  | y |  |  |  | y |  | 3 |  |
| *ARVC-03* | PRKAG2 | c.425C>T | p.Thr142Ile |  | |  |  | y |  |  |  |  |  | y |  |  |  |  |  | 3 |  |
| *ARVC-04* | PLN | c.40_42delAGA | p.Arg14del |  | | y | y | y | y |  |  |  |  | y |  |  |  |  |  | 5 |  |
|  | MYH6 | c.3607dupG | p.Ala1203Glyfs*30 |  | |  |  | y |  |  |  |  |  | y |  |  |  |  | y | 3 |  |
|  | TTN | c.59113C>T | p.Arg19705Cys |  | |  |  | y |  |  |  |  |  | y |  |  | y | y | y | 2 |  |
| *ARVC-05* | LMNA | c.1073A>G | p.Glu358Gly |  | |  |  | y |  | y |  |  | y | y |  |  |  |  |  | 4 |  |
|  | PKP2 | c.2326T>C | p.Ser776Pro |  | |  |  | y |  |  |  |  |  | y |  |  |  | y | y | 3 |  |
| *ARVC-06* | PKP2 | c.2146-1G>C |  | Y | |  | y | y |  |  |  |  |  | y | y |  |  |  |  | 5 |  |
|  | PKP2 | c.1138G>A | p.Glu380Lys |  | |  |  | y |  |  |  |  |  | y |  |  |  |  | y | 3 |  |
|  | RYR2 | c.4069G>A | p.Asp1357Asn |  | |  |  | y |  |  |  |  |  | y |  |  |  |  | y | 3 |  |
|  | TTN | c.102877A>G | p.Lys34293Glu |  | |  |  | y |  |  |  |  |  | y |  |  |  |  | y | 3 |  |
| *ARVC-07* | DES | c.1315G>A | p.Glu439LYS |  | |  |  | y |  |  |  |  |  | y | y |  |  |  |  | 3 |  |

**Abbreviations**: **ARVC**=arrhythmogenic right ventricular cardiomyopathy, **DCM**=dilated cardiomyopathy, **HNOCM**=hypertrophic non-obstructive cardiomyopathy, **LVNC**=left ventricular non-compaction cardiomyopathy, **MRI**=magnetic resonance imaging, **RCM**=restrictive cardiomyopathy, **y**=yes, meets criterion.

**^1^**For reference sequence see Tab.S1, the nomenclature of sequence variants based on the rules of the *Human Genome Variation Society* (HGVS) (http://www.hgvs.org)

**^2^**Criteria and classification according to the ACMG guidelines ([1](#_ENREF_1)): Pathogenicity classes: class 1, *benign*; class 2, *likely benign*; class 3, *uncertain significance*; class 4, *likely pathogenic*; class 5, *pathogenic*. The ACMG criterial ([1](#_ENREF_1)): **PVS1**, loss of function variant; **PS1**, known pathogenic amino acid change; **PS3**, functional studies support damaging effect; **PM2***, (adjusted) the allele frequency *cut-off* was set to a value ≤ 0,0005; **PM4**, in frame insertions/deletions; **PM5**, novel missense change at a position where a different missense change determined to be pathogenic; **PM6**, *de novo* variant without confirmation of paternity and maternity; **PP2**, missense variant in a gene in which missense variants are a common mechanism of disease; **PP3**, computational evidence; **PP4**, patient´s phenotype is highly specific for a disease with a single genetic etiology; **PP5**, the variant is recently reported as pathogenic, but the evidence is not available for independent evaluation; **BS2**, observed in a healthy adult individual; **BS4**, lack of segregation in affected family members; **BP4**, multiple lines of computational evidence suggest no impact on gene or gene product; **BP5**, variant found in a case with an alternate molecular basis for disease. All variants met basically the ACMG criteria PP4 and PM2.

**^3^**References for novel missense change at an amino acid residue where a different missense change determined to be pathogenic (ACMG criterion PM5) and novel null variants (nonsense, frameshift, canonical ±1 or 2 splice site) in a gene where loss-of-function is a known mechanism of disease (ACMG criterion PVS1), respectively. References for known variants are given in the main section of the manuscript.

**^4^**Affects only TTN-isoform N2BA.

**^5^**Affects only TTN-isoform N2B. Unmarked *TTN*-variants affects TTN-N2BA and TTN-N2B.

**^6^**See Results section in the main part of the manuscript.

**^7^**Cosegregation was used as stronger evidence (see note for ACMG criterion PP1).

**^8^**Younger sister with no disease symptoms in MRI.
