# Supplementary material for: High proportion of genetic cases in patients with advanced cardiomyopathy including a novel homozygous Plakophilin 2-gene mutation
Source: PLoS One. 2017 Dec 18;12(12):e0189489. doi: 10.1371/journal.pone.0189489 (PMC5734774; doi:10.1371/journal.pone.0189489)
Supplement: S7 Table — (DOCX) [file pone.0189489.s008.docx]

**S7 Table. *TTN*-gene-variants**

| **Nucleotide change** | **Amino acid change** | **Position**  **(Hg19)** | **TTN-Band** | **PSI %**^1^ |
| --- | --- | --- | --- | --- |
| c.25570G>A | p.Gly8524Arg^4^ | 179581891 | I-Band | 8 |
| c.65035_65036 delGC | p.Ala21679fs | 179449241 | A-Band | 100 |
| c.92595A>C | p.Leu30865Phe | 179413758 | A-Band | 100 |
| c.61682C>G | p.Ser20561X | 179454779 | A-Band | 100 |
| c.55745C>T | p.Pro18582Leu | 179465886 | A-Band | 100 |
| c.41486G>C | p.Gly13829Ala | 179500812 | I-Band | 100 |
| c.86637T>A | p.Asn28879Lys | 179424222 | A-Band | 100 |
| c.83062C>T | p.Arg27688Cys | 179427797 | A-Band | 100 |
| c.12438_12448 del11 | p.Ser4147fs^5^ | 179605511 | I-Band | 100 |
| c.87355delG | p.Ala29119fs | 179422725 | A-Band | 100 |
| c.54140C>T | p.Ala18047Val | 179469764 | A-Band | 100 |
| c.51436+1G>A |  | 179474816 | A-Band | 100 |
| c.11887G>A | p.Gly3963Arg^5^ | 179606073 | I-Band | 100 |
| c.42909_42910 delTG | p.Cys14303Trpfs | 179498175 | I-Band | 100 |
| c.521A>G | p.Tyr174Cys | 179665184 | Z-Band | 100 |
| c.54768 | p.Ser18258Valfs | 179468645 | A-Band | 100 |
| c.101774_101776 dupAAG | p.Glu33925dup | 179399565 | M-Line | 100 |
| c.106403T>A | p.Leu35468His | 179394815 | M-Line | 100 |
| c.74305A>G | p.Asn24769Asp | 179436554 | A-Band | 100 |
| c.59113C>T | p.Arg19705Cys | 179457733 | A-Band | 100 |
| c.102877A>G | p.Lys34293Glu | 179398465 | M-Line | 100 |

**^1^** PSI=percentage spliced in: a measure of splicing derived from RNAseq, this is an estimate of the percentage of TTN transcripts that incorporate a given exon. 100% implies a constitutively expressed exon. The data shown represent exon usage in the human left ventricle, derived from patients with DCM, see ([19](#_ENREF_19)) and <http://cardiodb.org/titin>. *TTN*-variant nomenclature refers to the longest predicted isoform (NM_001267550.1, NP_001254479.1).
