# Supplementary material for: High proportion of genetic cases in patients with advanced cardiomyopathy including a novel homozygous Plakophilin 2-gene mutation
Source: PLoS One. 2017 Dec 18;12(12):e0189489. doi: 10.1371/journal.pone.0189489 (PMC5734774; doi:10.1371/journal.pone.0189489)
Supplement: S8 Table — (DOCX) [file pone.0189489.s009.docx]

**S8 Table.** Genes listed in the Human Gene Mutation Database (HGMD^®^) Professional 2015.1 when using the search term ‘cardiomyopathy*’* (n=156).

| **Gene** |
| --- |
| *AARS2* |
| *ABCC9* |
| *ACTA1* |
| *ACTC1* |
| *ACTN2* |
| *ADH5* |
| *ADORA1* |
| *ALMS1* |
| *ANKRD1* |
| *BAG3* |
| *BRAF* |
| *CACNA1C* |
| *CALR3* |
| *CASQ2* |
| *CAV3* |
| *COX15* |
| *CRYAB* |
| *CSRP3* |
| *CTF1* |
| *CTNNA3* |
| *CHRM2* |
| *DES* |
| *DMD* |
| *DNAJC19* |
| *DOLK* |
| *DSC2* |
| *DSG2* |
| *DSP* |
| *ELAC2* |
| *ELN* |
| *EMD* |
| *EPHA4* |
| *FHL1* |
| *FHOD3* |
| *FKTN* |
| *FLNC* |
| *FLT1* |
| *FOXD4* |
| *FXN* |
| *GATA4* |
| *GATA5* |
| *GATA6* |
| *GATAD1* |
| *GLA* |
| *GTPBP3* |
| *HADHB* |
| *HCN4* |
| *HSPB7* |
| *ILK* |
| *INS-IGF2* |
| *ISL1* |
| *JPH2* |
| *JUP* |
| *KCNQ1* |
| *KLF10* |
| *LAMA2* |
| *LAMA4* |
| *LDB3* |
| *Idb3z4* |
| *LMNA* |
| *Imna2* |
| *LRRC10* |
| *MAP2K2* |
| *MIB1* |
| *MRPL3* |
| *MRPL44* |
| *MTO1* |
| *MURC* |
| *MYPBC3* |
| *MYH7* |
| *MYL2* |
| *MYL3* |
| *MYH7B* |
| *MYLK2* |
| *MYO6* |
| *MYOM1* |
| *MYOZ2* |
| *MYPN* |
| *NCOA6* |
| *NDUFAF1* |
| *NEBL* |
| *NEXN* |
| *NKX2-5* |
| *NPPA* |
| *OBSCN* |
| *PDLIM3* |
| *PKP2* |
| *PLN* |
| *POLG* |
| *PRDM16* |
| *PRKAG2* |
| *PRKCA* |
| *PSEN1* |
| *RAF1* |
| *RANGRF* |
| *RBCK1* |
| *RBM20* |
| *RYR2* |
| *SCN5A* |
| *SGCA* |
| *SGCD* |
| *SGCG* |
| *SLC25A3* |
| *SLC25A4* |
| *SOS1* |
| *SPEG* |
| *SRI* |
| *SYNE1* |
| *SYNM* |
| *TAX1BP3* |
| *TAZ* |
| *TBX20* |
| *TBX5* |
| *TCAP* |
| *TCF21* |
| *TMEM43* |
| *TMEM70* |
| *TMPO* |
| *TNNC1* |
| *TNNI3* |
| *TNNI3K* |
| *TNNT2* |
| *tnnt2i1* |
| *TOR1AIP1* |
| *TPM1* |
| *tpm17* |
| *TRIM54* |
| *TRIM55* |
| *TRIM63* |
| *TSFM* |
| *TTN* |
| *ttnltv* |
| *ttntvn2b* |
| *TTR* |
| *TXNRD2* |
| *VCL* |
| *VPS13A* |
| *XDH* |
| *ADORA3* |
| *ADRA2B* |
| *AGK* |
| *AKAP9* |
| *ALDH3A2* |
| *ARFGEF2* |
| *BMP10* |
| *C5* |
| *CACNA2D4* |
| *CACNG1* |
| *CADPS* |
| *CALM3* |
| *COA5* |
| *COA6* |
| *LAMP2* |
| *MYH6* |
| *NDUFB11* |
| *TGFB3* |
