# Supplementary material for: High proportion of genetic cases in patients with advanced cardiomyopathy including a novel homozygous Plakophilin 2-gene mutation
Source: PLoS One. 2017 Dec 18;12(12):e0189489. doi: 10.1371/journal.pone.0189489 (PMC5734774; doi:10.1371/journal.pone.0189489)

**S1 Figure. Age of patients at the initial diagnosis**. The mean age at diagnosis was not significantly different in DCM- (31±17 years) and ARVC-cases (35±17 years). Mean age at diagnosis in RCM 11±9 years.


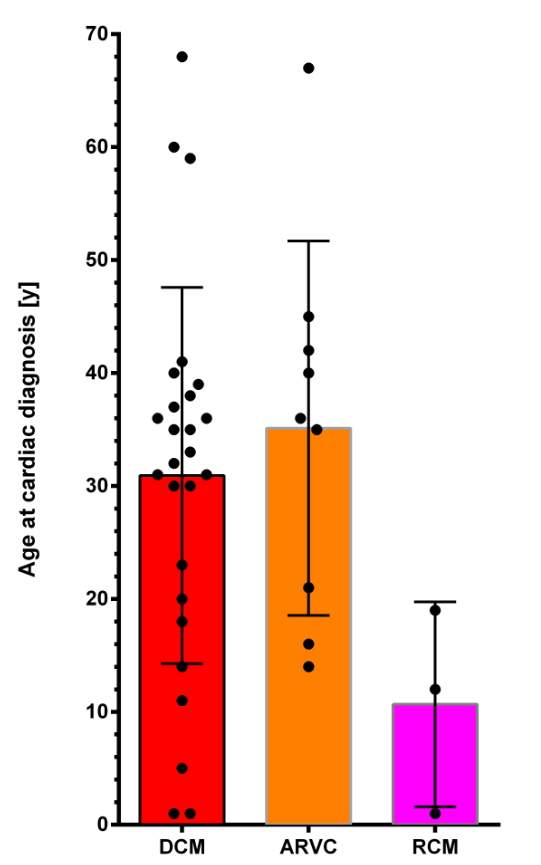

Supplement: S1 Fig — (DOCX) [file pone.0189489.s010.docx]
