# Supplementary material for: High proportion of genetic cases in patients with advanced cardiomyopathy including a novel homozygous Plakophilin 2-gene mutation
Source: PLoS One. 2017 Dec 18;12(12):e0189489. doi: 10.1371/journal.pone.0189489 (PMC5734774; doi:10.1371/journal.pone.0189489)
Supplement: S2 Fig — (DOCX) [file pone.0189489.s011.docx]

**S2 Figure. Family histories and variant co-segregation**

Pedigrees of the families mentioned in the main part of the manuscript. Squares represent males and circles females. Deceased individuals are indicated by slashes. Filled symbols indicate individuals affected with the family typical cardiomyopathy (DCM, RCM, and ARVC). Striped symbols indicate individuals with evidence for heart disease as reported by the family. Grey filled symbols indicate individuals with a suspected clinical phenotype. The index-patient is marked with an arrow. Genotypes are shown by (+) or (-) (presence or absence of the gene mutation, respectively).

**Abbreviations**: **HTx**=heart transplantation, **SCD**=sudden cardiac death, **TAH**=total artificial heart. For family details see main section of the manuscript.


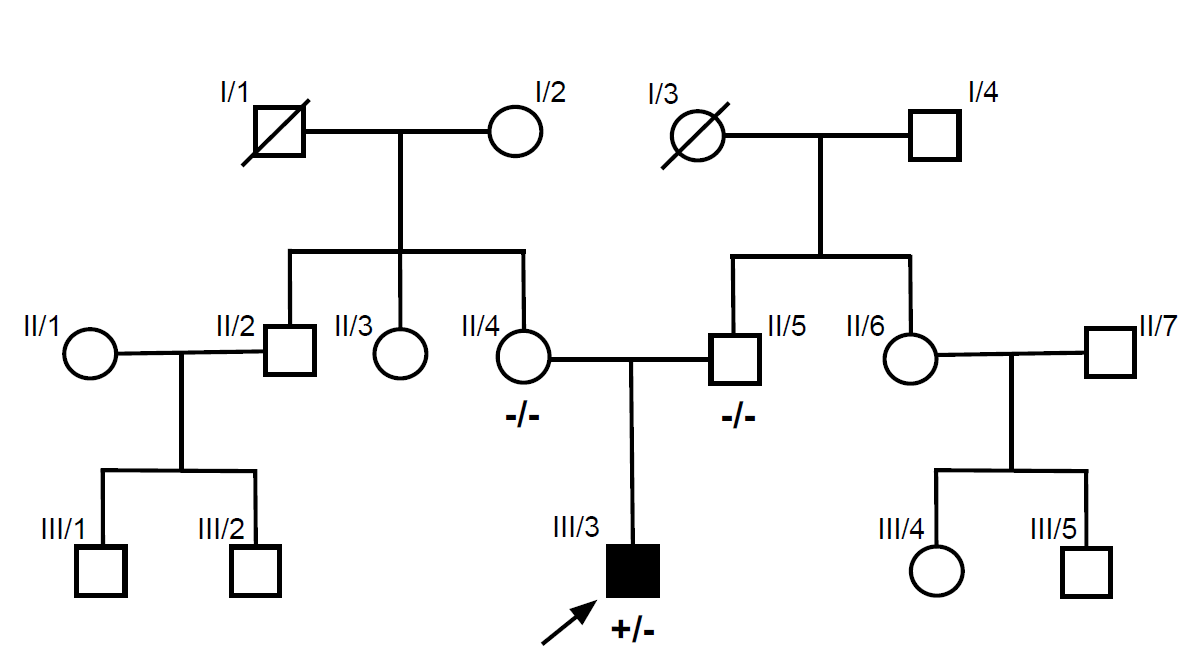


**Pedigree of family DCM-01**. Segregation of the class 4 *de novo* mutation *MYH7* c.5390T>C, p.(Leu1797Pro) is shown. Patient **DCM-01** (III/3, Fig.S2) had already clinical symptoms of heart disease at the age of one year and underwent ventricular assistant device (VAD) implantation and HTx aged 13 years. He is the only child of clinically unaffected parents at present aged 46 and 48 years, respectively. No family history of cardiac disease was reported. Two *likely pathogenic* (class 4) heterozygous missense mutations were identified in this patient. The mutation in the gene encoding for the myosin regulatory light chain (*MYL2* c.401A>C, p.Glu134Ala) was described once in a HCM-index patient ([25](#_ENREF_25)). Functional evidence comes from *in vitro* data that indicates lower energy conversion efficiency in the muscle fibers compared to the wild type protein ([26](#_ENREF_26)). In accordance to the classification previously made by others ([57](#_ENREF_57)) we classified this variant as a class 4 mutation (Tab.3). This classification, however, is preliminary since the clinically unaffected father (II/5) is also carrier of the mutation. Therefore, it is currently still unknown whether this is an indication of incomplete or age dependent penetrance or missing impact for the onset of the disease. The second variant is a novel *de novo* mutation in the *MYH7*-gene (c.5390T>C, p.Leu1797Pro) that is a known cardiomyopathy disease gene with numerous pathogenic missense variants ([7](#_ENREF_7)). In this part of the molecule different mutations destabilizing the coiled-coil were previously reported ([58](#_ENREF_58), [59](#_ENREF_59)) whereas Homburger *et al.* recently revealed a region of increased genetic tolerance in this part of the protein ([60](#_ENREF_60)). The coiled-coil of myosin-7 is stabilized by a hydrophobic seam ([61](#_ENREF_61)) caused by a heptad repeat of amino acids. Leu1797 is located at position 'a' of the heptad which is important for rod assembly and stability ([58](#_ENREF_58)). In addition, the proline residue at codon 1797 might be functionally critical, since it is located within the coiled-coil region of myosin heavy chain and might cause an interruption of the alpha helix, since this iminoacid cannot form H-bonds. We classified this variant as a class 4 mutation (Tab.3).


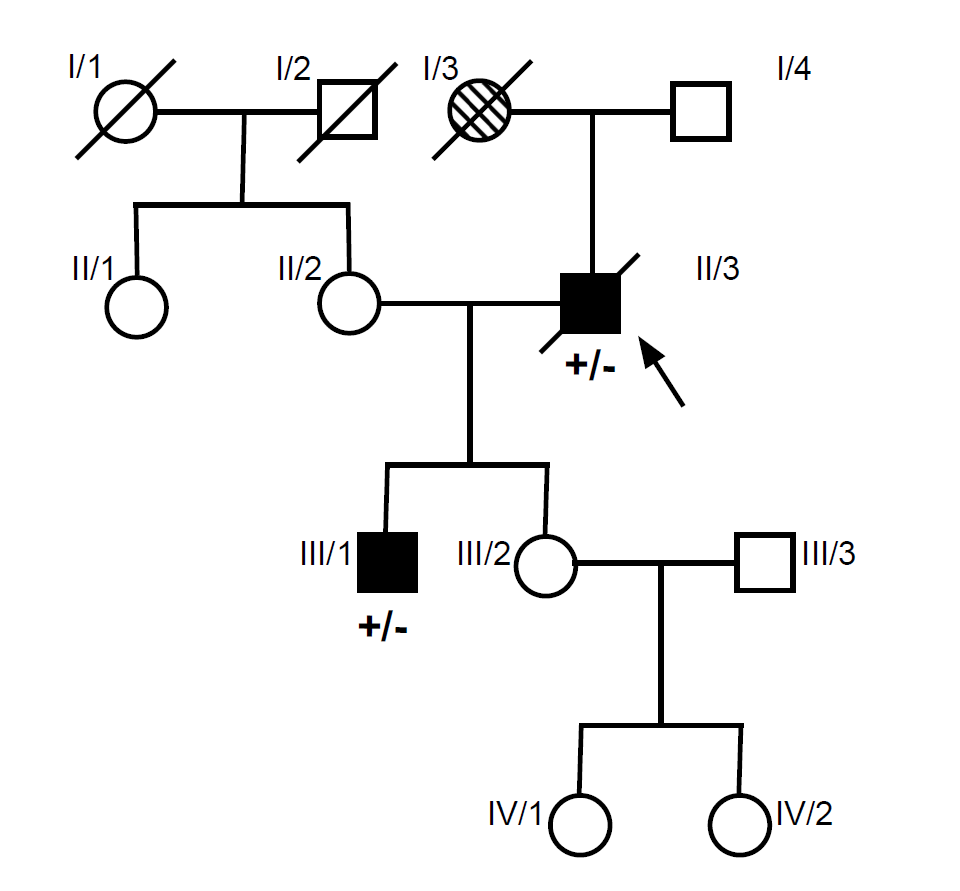


**Pedigree of family DCM-03.** Segregation of the class 4 variant *TTN* c.65035_65036delGC, p.(Leu21679fs) is shown. From patient **DCM-03** (II/3, Fig.S3) the time of disease onset was not available but an ICD implantation at the age of 68 years may indicate midlife or late onset of the disease. He is carrier of 3 heterozygous variants in the genes *TTN* and *DSC2*. Titin is affected by two variants (c.65035_65036delGC, p.Leu21679Leufs*6, and c.92595A>C, p.Leu30865Phe) that are thought to affect the same allele because the patient’s son (III/1) is also heterozygous for both variants and it seems to be unlikely that the genetically untested healthy mother (II/2) of the son is by chance carrier of one of the *TTN*-gene variants. Accordingly, the missense variant is located down-stream of the truncating variant and thus might be non-functional. The *TTN*-gene truncating variant was classified as *likely pathogenic* whereas the missense variant is of *uncertain significance* (Tab.3). The clinical presentation of the son (III/1) matches with the characteristics of *TTN*-tv that are reported to be associated with a penetrance of 95% at ages more than 40 years ([18](#_ENREF_18)). He was diagnosed with DCM (NYHA I) with a LVEDD of 66 mm and reduced ejection fraction (45%) at the age of 41 years. The *DSC2*-variant c.1307G>T, p.Gly436Val did not co-segregate in the son and was classified as *likely benign* taken into account that it might be a possible modifier. The clinically unaffected daughter (III/2) who is 4 years older than her brother refused genetic testing.


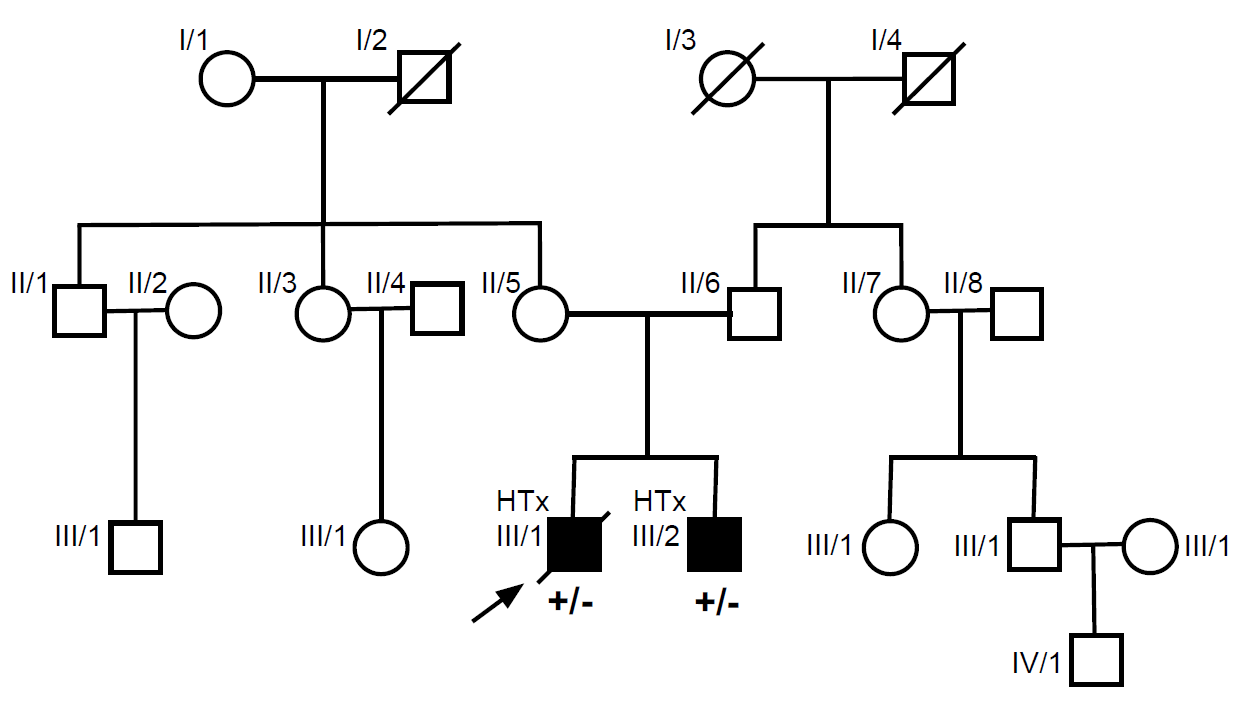


**Pedigree of family DCM-05.** Segregation of the class 4 variant *TNNC1* c.435C>A, p.(Asp145Glu) is shown. **DCM-05** (III/1, Fig.S4) is a young man who deceased 13 years after HTx at the age of 14 years. His 3 years older brother (III/2) underwent HTx at the age of 11 years. Both brothers are carriers of two *TNNC1-*variants (Tab. 3) in a compound heterozygous state as determined by cloning and sequencing of *long-range* PCR fragments*.* Class 4 mutation c.435C>A, p.Asp145Glu was previously identified in HCM- and DCM-patients ([62](#_ENREF_62), [63](#_ENREF_63)). Based on *in vitro* functional data an altered Ca^2+^ binding function was suggested that contributes to disease associated dysfunction(s) of the myofilament ([29](#_ENREF_29), [30](#_ENREF_30)). The second *TNNC1*-gene variant (c.184G>A, p.Asp62Asn) is novel and of *uncertain significance* (class 3) considering the ACMG criteria. So far, no cardiological and genetic examinations of the parents or further family members were performed. Therefore, it remains unclear whether a single *TNNC1*-variant or the compound heterozygous genotype is related to the severe disease phenotype of the brothers.


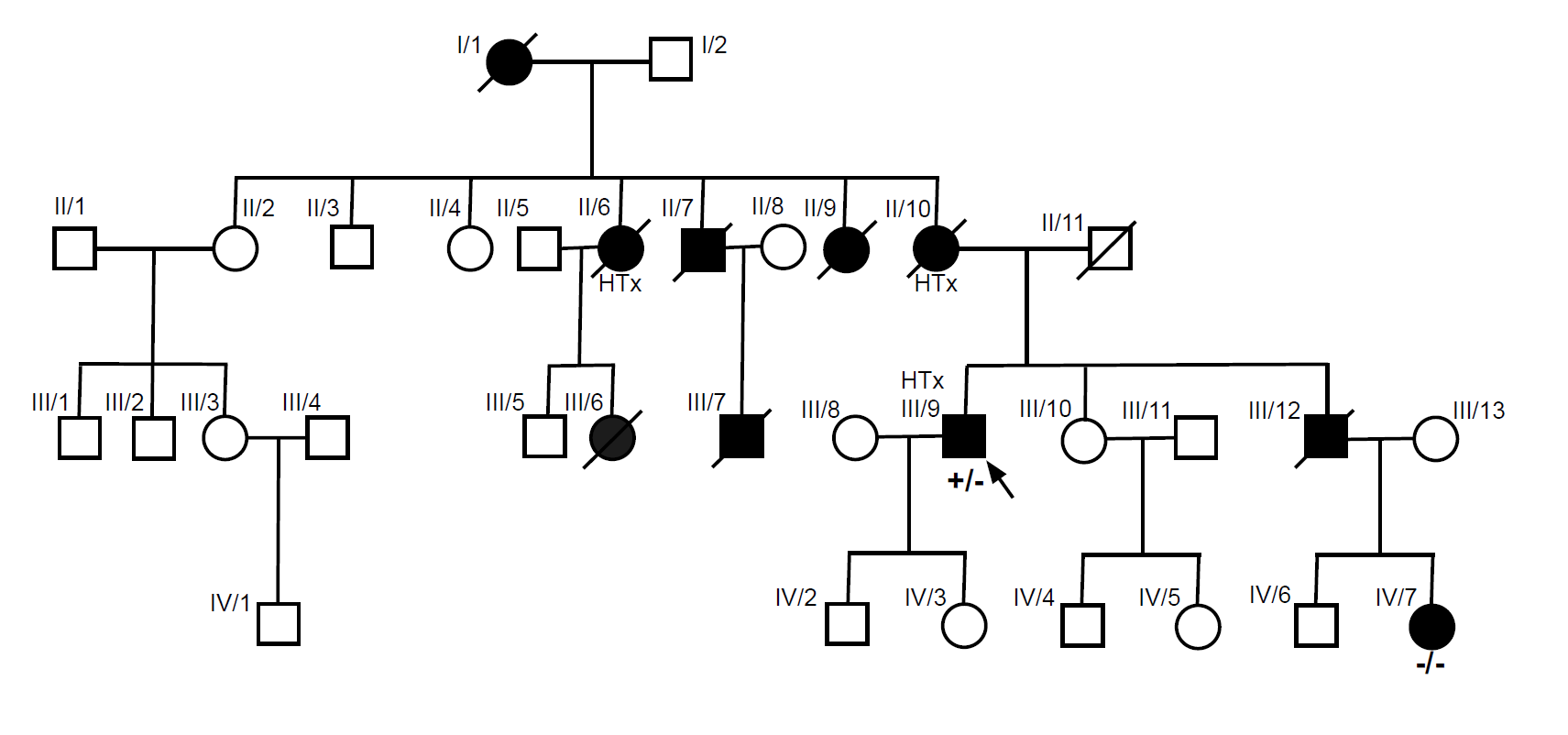


**Pedigree of family DCM-10**. Segregation of the class 2 variant *DSP* c.3551G>A, p.(Arg1184Gln) is shown. The mother (II/10) and one aunt (II/6) of patient **DCM-10** (III/9, Fig.S5) underwent HTx. Both are already deceased, his mother died at the age of 48 years. His brother (III/12) diagnosed with DCM at the age of 38 years died on the HTx waiting list. A niece (IV/7) was diagnosed with DCM at the age of 18 years. The identified *DSP*-variant c.3551G>A, p.Arg1184Gln did not co-segregate with the disease and was classified as *likely benign* (class 2; Tab.3). This variant might have no or only a minor effect on disease development and thus the genetic cause of the familial DCM remains to be elucidated.


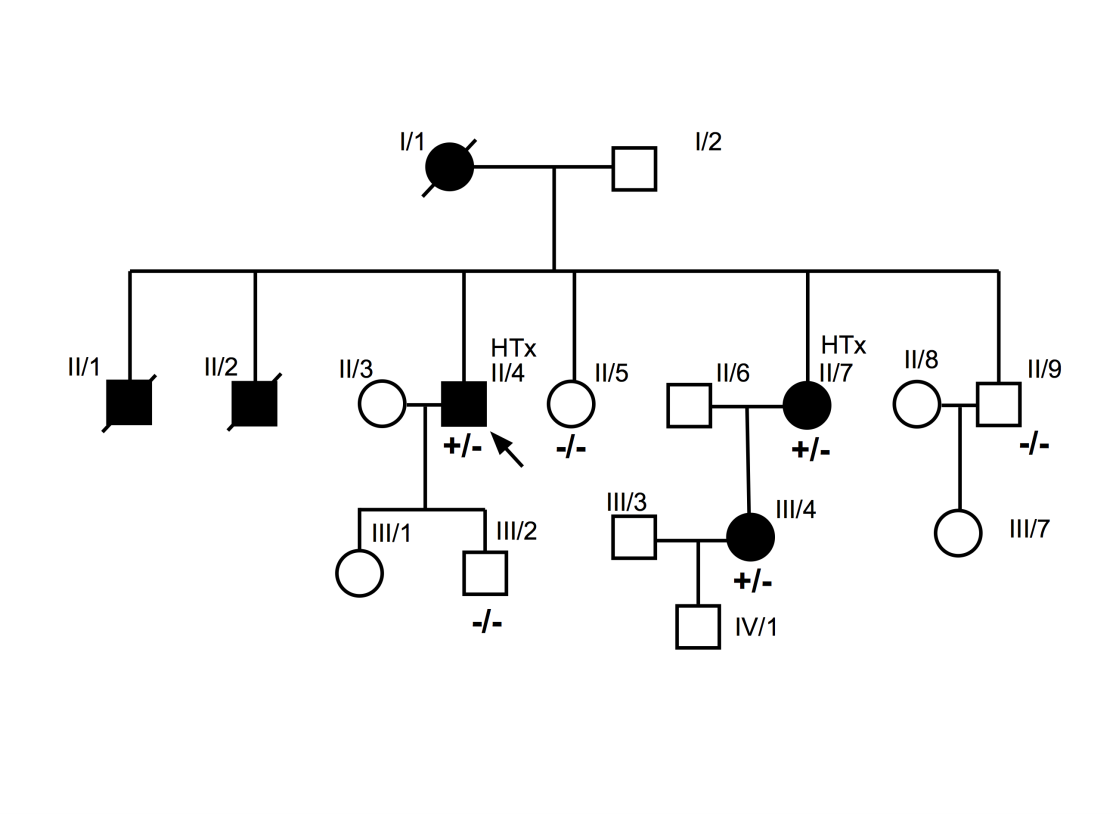


**Pedigree of family DCM-14**. Segregation of the class 4 variant *RBM20* c.1904C>G p.(Ser635Cys) is shown. In patient **DCM-14** (II/4, Fig.S6) with obvious evidence of familial DCM the missense mutation c.1904C>G, p.Ser635Cys in *RBM20* encoding the RNA-binding motif protein 20 (RBM20) was identified (Tab.3). The substitution p.Ser635Ala has previously been reported to be associated with severe cardiomyopathy ([36](#_ENREF_36), [64](#_ENREF_64)). Of note, *in vitro* analysis of the mutant protein revealed deviating characteristics for RBM20 p.Ser635Cys compared to the wild type protein (data not shown). The mother (I/1, 59 years) and two brothers (34 and 26 years, respectively; II/1, II/2) of patient DCM-15 deceased from heart disease. One brother died while waiting for HTx. A sister (II/7) with DCM underwent HTx aged 32 years. Two other siblings (brother 54 years (II/9), sister 43 years (II/5)) have no signs of heart disease. The heart transplanted sister (II/7) and her diseased (proofed by ECG and TTE) daughter (III/4), as well as the two healthy siblings (II/5, II/9) were available for co-segregation studies. *RBM20* c.1904C>G, p.Ser635Cys co-segregates with the disease. *RBM20* c.1904C>G, p.Ser635Cys was classified as a class 4 mutation. A second variant in *DSP* (c.3616T>A, p.Leu1206Ile) was identified in all genetically tested family members and was classified as class 3.


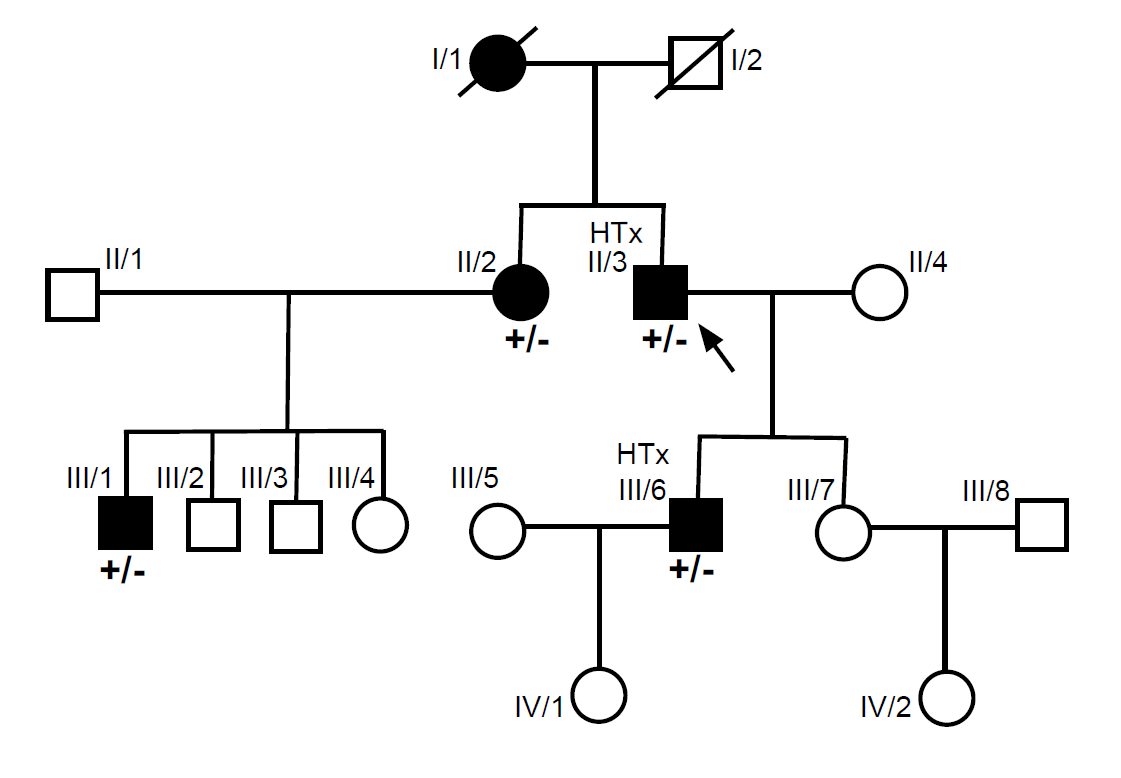


**Pedigree of family DCM-15**. Segregation of the class 5 variant *RBM20* c.1913C>T, p.(Pro638Leu) is shown. The *RBM20* mutation c.1913C>T, p.Pro638Leu (class 5) was found in HTx-patient **DCM-15** (II/3, Fig.S7). His son (III/6) was transplanted at the age of 33 years. His sister (II/2) was diagnosed with DCM at the age of 36 years, her son (III/1) was diagnosed with DCM at the age of 16 years and received a *ventricular assistance device* at the age of 21 years. The *RBM20* mutation co-segregated with disease and was identified in the patient´s son (III/6), his sister (II/2) and his nephew (III/1) while a second *TTN* c.54140C>T, p.Ala18047Val missense variant (class 3) was only present in the index patient (II/3) and his son (III/6). *RBM20* c.1913C>T, p.Pro638Leu is a known DCM-associated mutation ([64](#_ENREF_64)) with altered splicing activity indicated by the results of an *in vitro* splice assay ([36](#_ENREF_36)) using highly homologous rat protein.


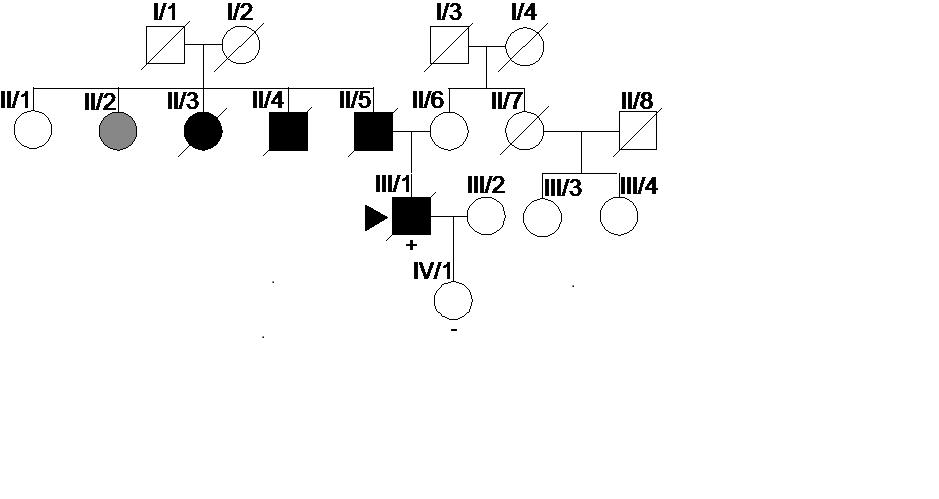


**SCD**

**SCD**

**/-**

**-/**

**SCD**

**Pedigree of family DCM-17**. Segregation of the class 5 variant *DES c.493_520delinsGCGT* is shown. Index-patient **DCM-17** (III/2, Fig.S8) is carrier of a heterozygous deletion/insertion (p.Gln165_Ala174delinsAlaSer) in the *DES*-gene that was classified as *pathogenic* (class 5). He presents with a myopathy and DCM as reported in association with desmin mutations ([65](#_ENREF_65)). His genetically untested father (II/5), a paternal uncle (II/4), and one aunt (II/3) died by SCD aged 44, 55, and 63 years, respectively, with unreported accompanying myopathy. His 30 years old healthy daughter (IV/1) is not carrier of the mutation.


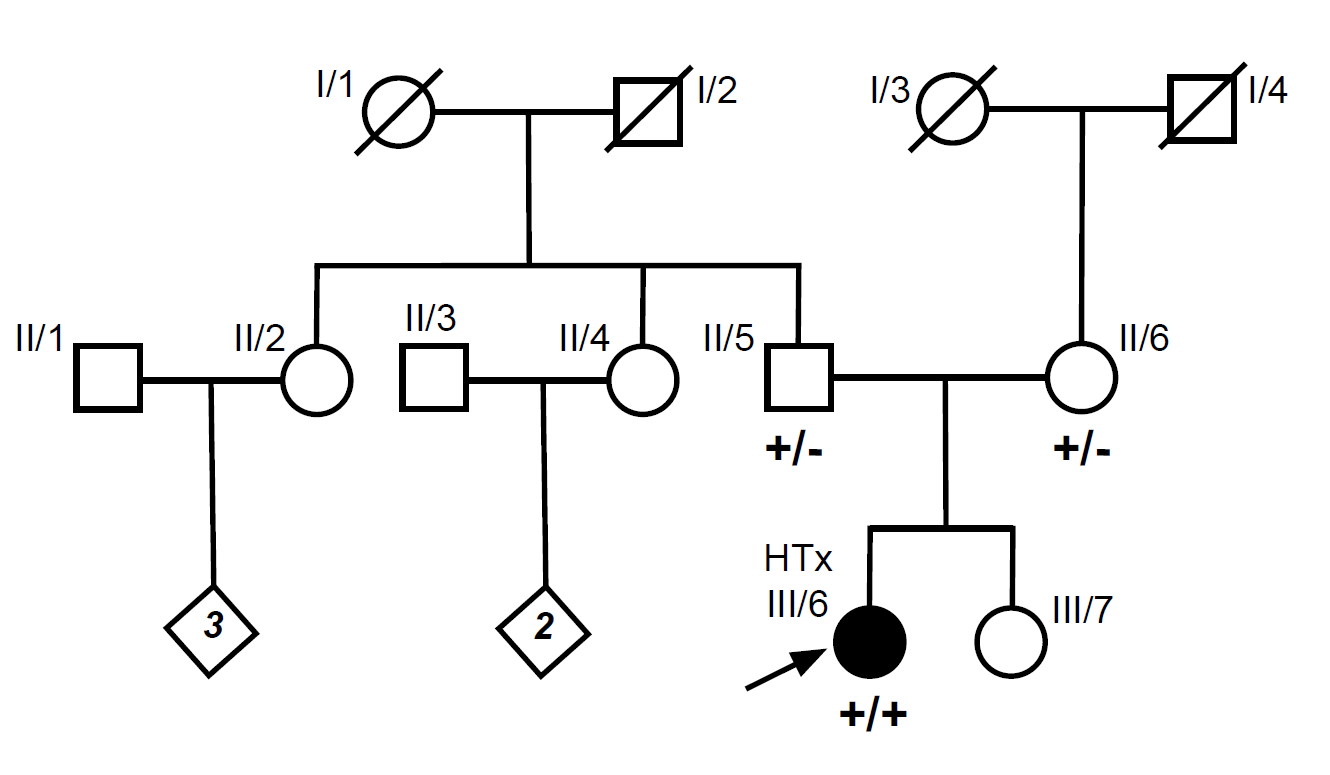


**Pedigree of family RCM-01**. Segregation of the class 3 variant *MYL3* (c.461G>A, p.(Arg154His) is shown. **RCM-01** (III/6, Fig.S9) is a homozygous carrier of a variant in the *MYL3-*gene (c.461G>A, p.Arg154His) encoding the essential myosin light chain. This variant has already been described in a young HCM-patient ([42](#_ENREF_42)). In addition, the substitution of arginine by cysteine at codon 154 of *MYL3* was reported in a patient with HCM ([57](#_ENREF_57)). Functional *in vitro* analysis revealed a lower affinity of myosin light chain 3 (MYL3) p.Arg154His to the lever arm of myosin-7 and therefore a lower functionality is expected ([43](#_ENREF_43)). The healthy unrelated parents (II/5, II/6, aged 44 and 49, respectively) of RCM-01 are each heterozygous for the variant. Since both previous studies ([42](#_ENREF_42), [57](#_ENREF_57)) provided no further information on the variant haplotype or a valid co-segregation, respectively, essential data are missing to decide whether this variant is pathogenic (if at all) only in the homozygous state. Furthermore, it remains to be determined whether the functional effect measured *in vitro* might lead to conclusions on the clinical phenotype *in vivo*. For this reason we classified the variant as *variant of uncertain significance* (class 3) although the ACMG guidelines might allow a classification with a stronger evidence for a possibly pathogenic effect.


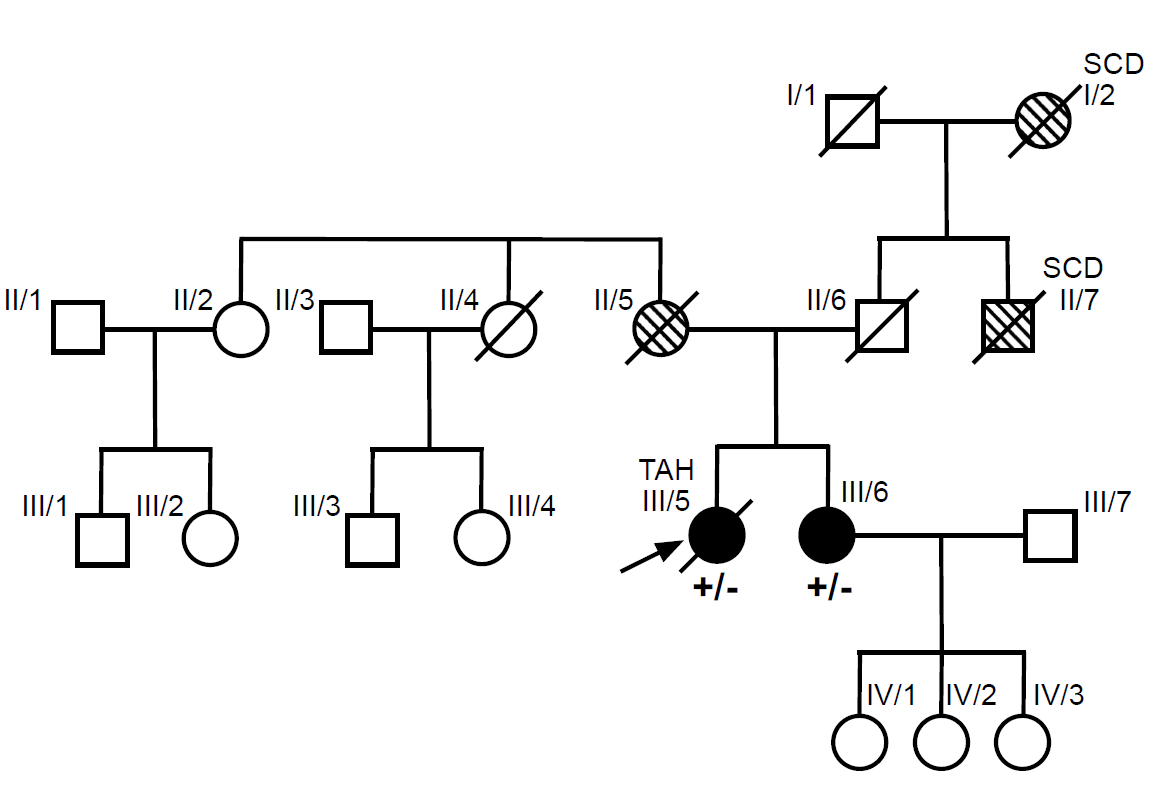


**Pedigree of family ARVC-04.** Segregation of the class 5 variant *PLN* c.40_42delAGA, p.(Arg14del) is shown. In **ARVC-04** (III/5, Fig.S10) who died at the age of 55 years while waiting for a donor heart, we identified the pathogenic *PLN*-gene mutation c.40_42delAGA, p.Arg14del (class 5). This in frame deletion is a well-documented founder mutation, which is frequent in The Netherlands ([66](#_ENREF_66)). The clinical phenotype is DCM in 66% and ARVC in 33% of cases. There are multiple reports available on the co-segregation and functional data ([48](#_ENREF_48), [49](#_ENREF_49), [67](#_ENREF_67), [68](#_ENREF_68)). In the paternal side of the family SCD of the grandmother (I/2) and one uncle (II/7) were reported. Her mother (II/5) who died at the age of 90 years had a dilated left ventricle as reported by the family. Her 55 years old sister (III/6) was diagnosed with borderline DCM and a preventive care with an ICD was recommended. The sister (III/6) also carried the *PLN*- and an unknown *MYH6*-gene variant (c.3607dupG, p.Ala1203Glyfs*30, class 3), while a *TTN*-missense variant (c.59113, p.Arg19705Cys, class 2) was only present in the index-patient.

Segregation studies of those variants that have not been described in detail were not performed because no additional family members were available, or further investigations were declined by the family or clinical geneticists, especially in cases of predictive testing.
