# Supplementary material for: High proportion of genetic cases in patients with advanced cardiomyopathy including a novel homozygous Plakophilin 2-gene mutation
Source: PLoS One. 2017 Dec 18;12(12):e0189489. doi: 10.1371/journal.pone.0189489 (PMC5734774; doi:10.1371/journal.pone.0189489)

**S3 Figure. Age of patients at the initial diagnosis versus variant classification**. The median age at diagnosis of DCM- and ARVC-cases (33 and 36 years, respectively) was not significantly different in dependence of variant classification.


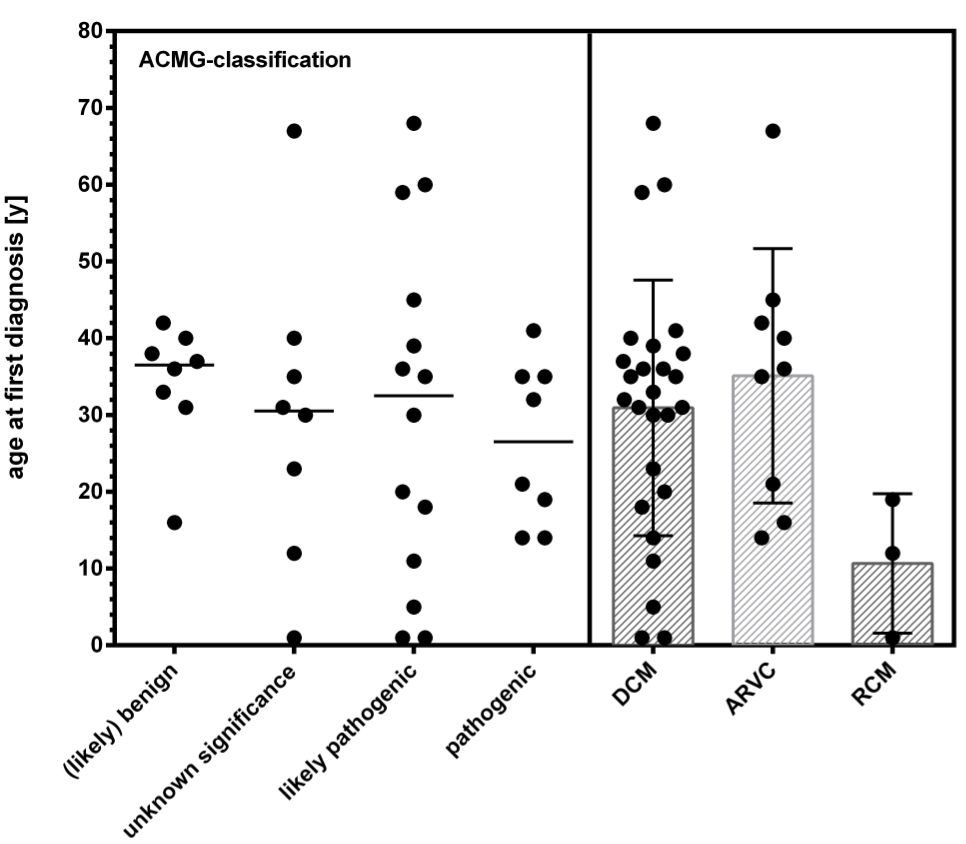

Supplement: S3 Fig — (DOCX) [file pone.0189489.s012.docx]
