# Supplementary material for: High proportion of genetic cases in patients with advanced cardiomyopathy including a novel homozygous Plakophilin 2-gene mutation
Source: PLoS One. 2017 Dec 18;12(12):e0189489. doi: 10.1371/journal.pone.0189489 (PMC5734774; doi:10.1371/journal.pone.0189489)

**S4 Figure**. **Number of *TTN*-variants in DCM patients compared to other cardiomyopathy**.

In DCM-patients *TTN*-variants were more frequent (p<0.05), whereas *TTN*-tv were increased by trend (p=0.05) when compared to the other cardiomyopathies of the study cohort.


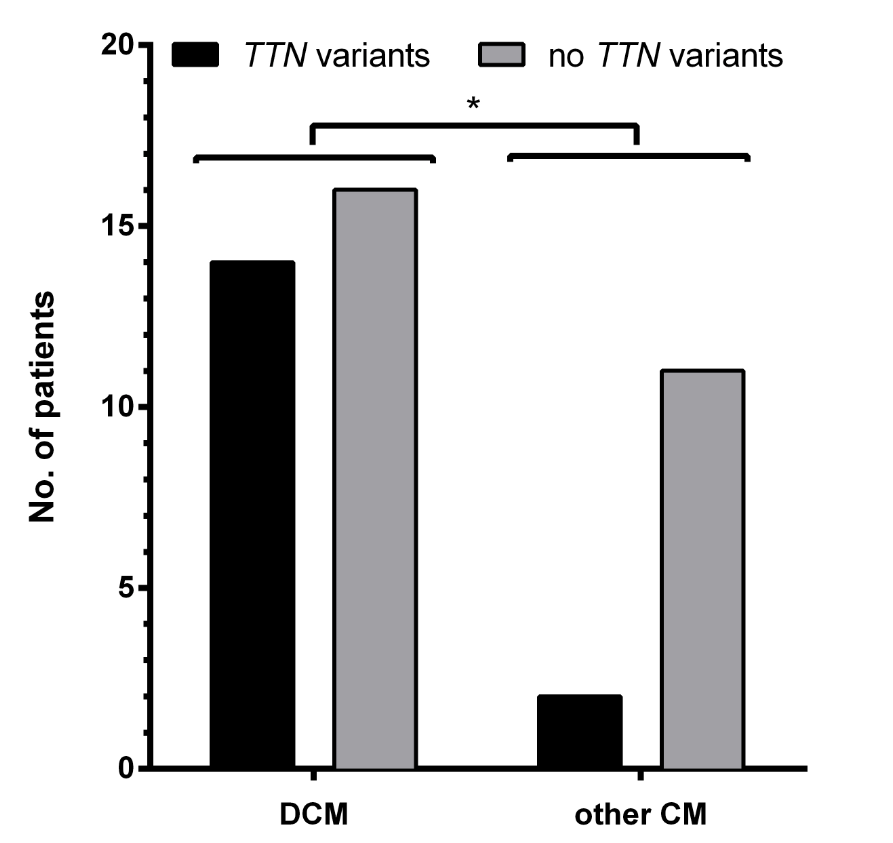

Supplement: S4 Fig — (DOCX) [file pone.0189489.s013.docx]
