# Supplementary material for: High proportion of genetic cases in patients with advanced cardiomyopathy including a novel homozygous Plakophilin 2-gene mutation
Source: PLoS One. 2017 Dec 18;12(12):e0189489. doi: 10.1371/journal.pone.0189489 (PMC5734774; doi:10.1371/journal.pone.0189489)
Supplement: S5 Fig — (DOCX) [file pone.0189489.s014.docx]

**S5 Figure**. **Electrocardiogram (ECG, 12-leads) of patients III/1 and III/10 of family DCM-23 with *PKP2* p.His679Tyr**. **A**. Patient III/1 (male): The ventricular heart rate is 71 bpm in sinus rhythm. In chest wall leads loss of R waves in V4, V5, and V6 and left precordial repolarization disorder indicated by T-wave flattening. **B**. Patient III/10 (female): The ventricular heart rate is 87 bpm in sinus rhythm. In chest wall leads the S-waves are deepened in V2-V3, slight ST-segment elevation in V2, V5, V6, lowered R-waves in V5 and V6, and slight left precordial repolarization disorder indicated by T-wave flattening.


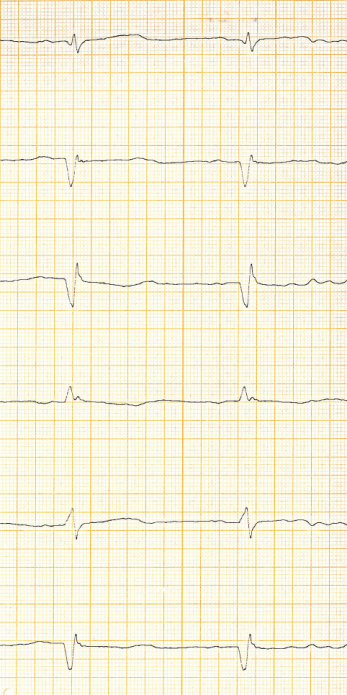

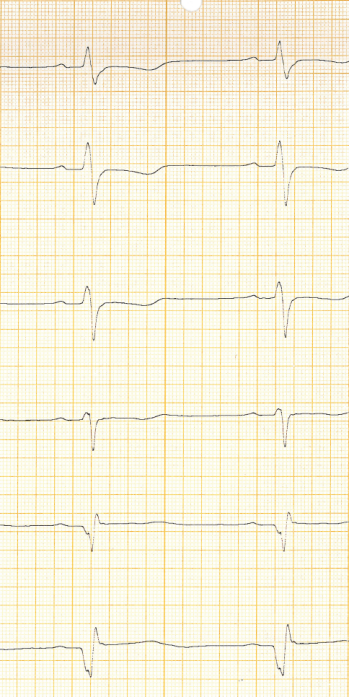

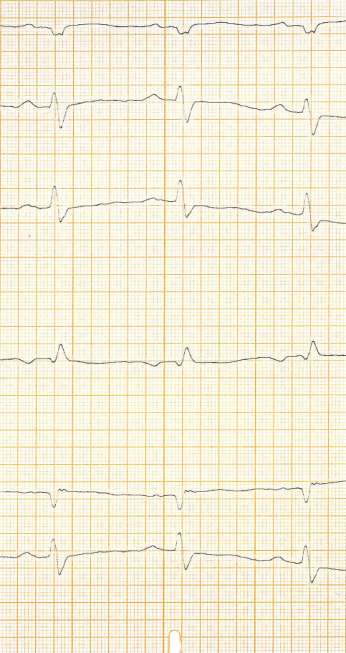

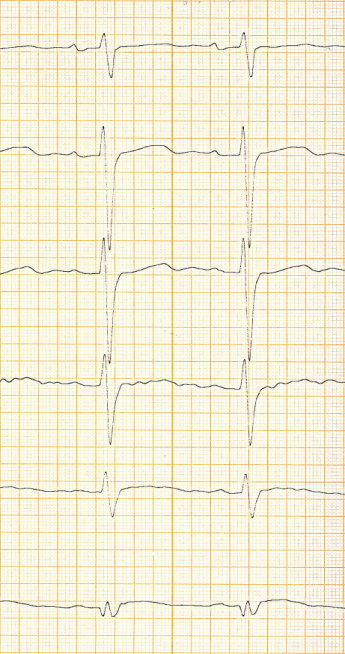


**I**

**II**

**III**

**aVR**

**aVL**

**aVF**

**V1**

**V2**

**V3**

**V4**

**V5**

**V6**

**I**

**II**

**III**

**aVR**

**aVL**

**aVF**

**V1**

**V2**

**V3**

**V4**

**V5**

**V6**

**A**

**B**
