# Supplementary material for: High proportion of genetic cases in patients with advanced cardiomyopathy including a novel homozygous Plakophilin 2-gene mutation
Source: PLoS One. 2017 Dec 18;12(12):e0189489. doi: 10.1371/journal.pone.0189489 (PMC5734774; doi:10.1371/journal.pone.0189489)
Supplement: S6 Fig — (DOCX) [file pone.0189489.s015.docx]

**S6 Figure. Quality score distribution of whole exome sequencing data of family DCM-23**. x-axis=Mean sequence quality (Phred score), y-axis=Number of sequences. For sample assignment see pedigree in the main part of the manuscript.

III/8

III/4

**
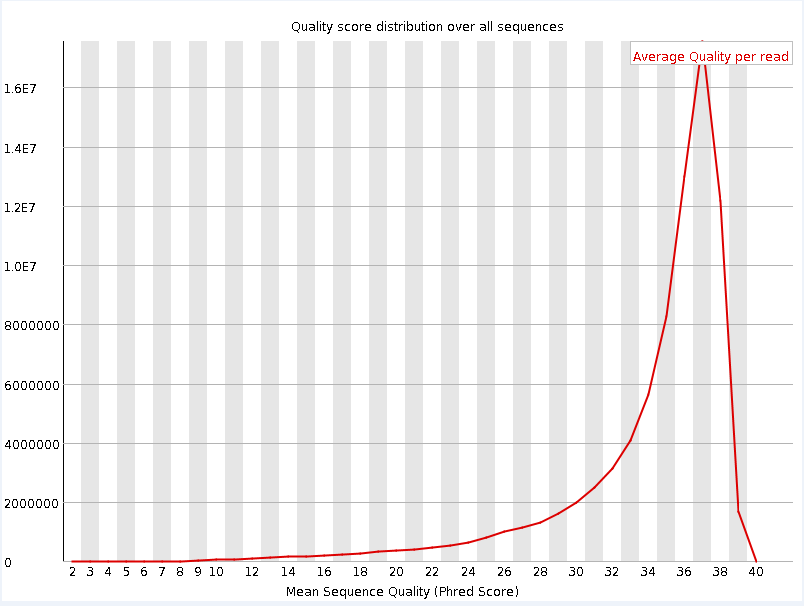

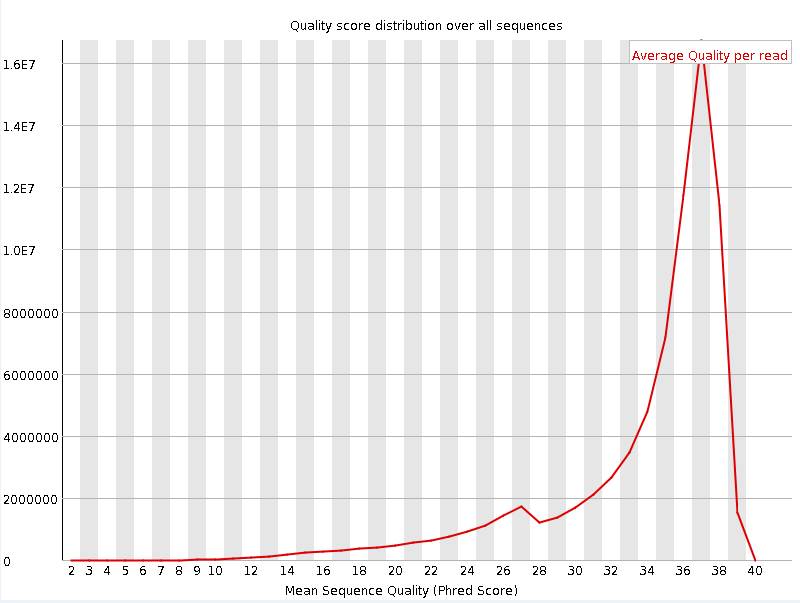
**

II/9

II/8

**
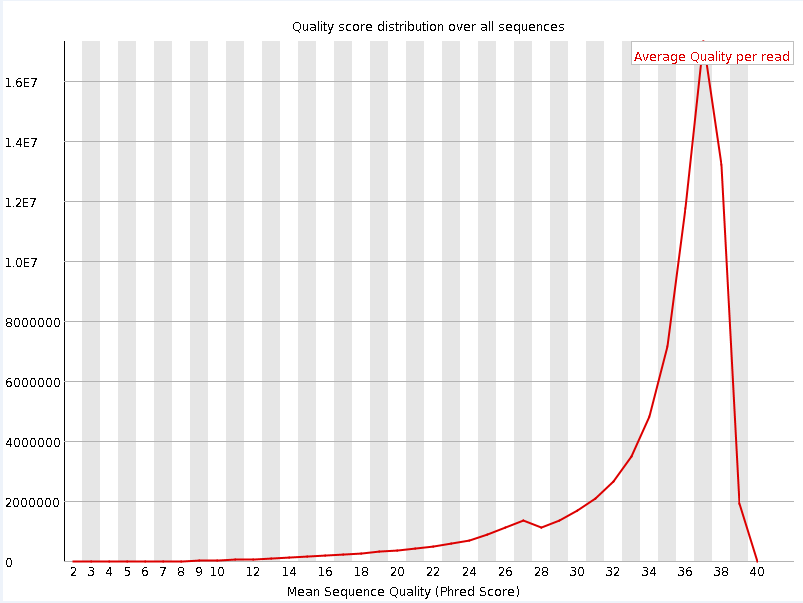

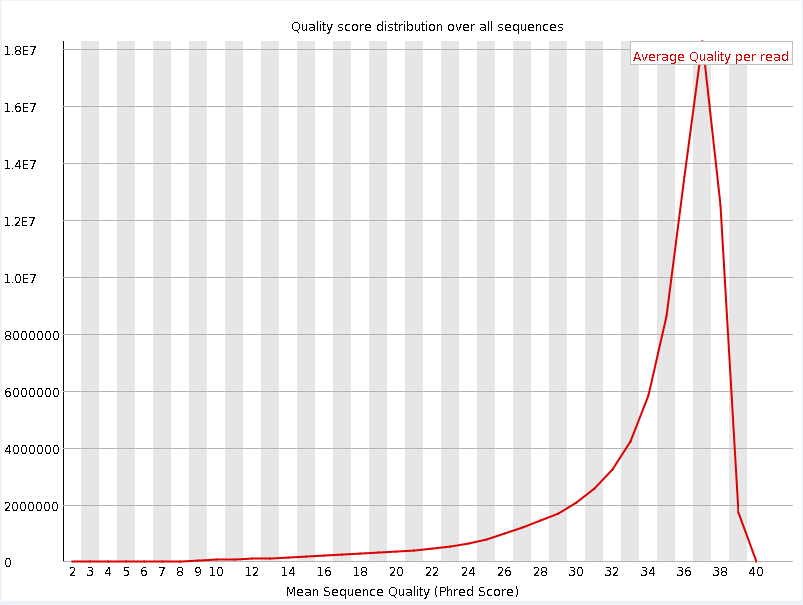
**
