# Supplementary material for: High proportion of genetic cases in patients with advanced cardiomyopathy including a novel homozygous Plakophilin 2-gene mutation
Source: PLoS One. 2017 Dec 18;12(12):e0189489. doi: 10.1371/journal.pone.0189489 (PMC5734774; doi:10.1371/journal.pone.0189489)

**S7 Figure.** **Real-time reverse transcription polymerase chain reaction of *PKP2*-mRNA**. Data given as relative expression in comparison to the “house keeping gene” glycerine-aldehyde-3-phosphate dehydrogenase (GAPDH). Gene expression was measured in heart tissue from explanted myocardium from 5 dilated cardiomyopathy (DCM)- , 5 arrhythmogenic right ventricular cardiomyopathy (ARVC)-patients , and 10 non-failing (NF) donor myocardium derived from rejected donor hearts (provided by Euro Heart Valve Bank, Rotterdam, The Netherlands). Expression of PKP2-mRNA was compared to gene expression measured in explanted heart tissue from 2 homozygous PKP2 p.H679Y variant carriers of family DCM-23 (female III/10, male III/1, for detail see family pedigree in the main section of the manuscript). All analyses were done 4 times, 5 NF donor myocardium analyses were done in duplicates. Expression of PKP2-mRNA between NF, DCM, ARVC and PKP2 p.H679Y was not significantly different. Affected male given as square, female as black dot.


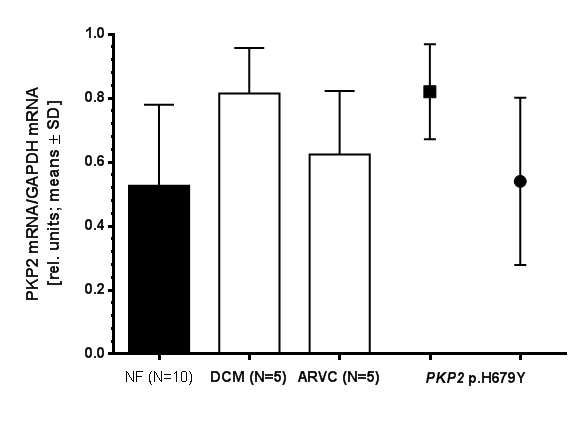

Supplement: S7 Fig — (DOCX) [file pone.0189489.s016.docx]
