# Supplementary material for: High proportion of genetic cases in patients with advanced cardiomyopathy including a novel homozygous Plakophilin 2-gene mutation
Source: PLoS One. 2017 Dec 18;12(12):e0189489. doi: 10.1371/journal.pone.0189489 (PMC5734774; doi:10.1371/journal.pone.0189489)

**S8 Figure**. **Western blot against PKP2 and GAPDH**.

**(A)** 10 µg total protein isolated from heart tissue from explanted myocardium from 4 unrelated dilated cardiomyopathy (DCM)-patients, 3 rejected non-failing (NF) donor hearts (provided by Euro Heart Valve Bank, Rotterdam, The Netherlands), and the explanted heart of a homozygous female PKP2 p.H679Y mutation carrier of family DCM-23 (III/10, for detail see family pedigree in the main section of the manuscript), respectively, were loaded onto the gel, blotted, and hybridized with PKP2a- and GAPDH- antibodies, respectively. Analysis of the PKP2 p.His679Tyr sample was done in triplicates. PKP2a (92,7 kDa) and GAPDH (35,9 kDa) protein bands are marked. **(B)** PKP2 quantity is given relative to GAPDH. The amount and run pattern of PKP2 were not significantly different between NF (n=3), DCM (n=4), and PKP2 p.H679Y (triplicate) carrier.

**A**.

20kDa

25kDa

37kDa

50kDa

75kDa

100kDa


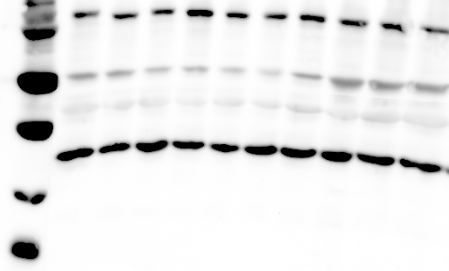


PKP2 (93kDa)

GAPDH (36kDa)

NF

DCM

p.H679Y

**B.**

.
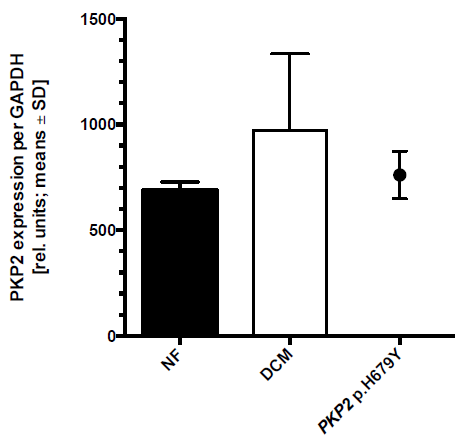

Supplement: S8 Fig — (DOCX) [file pone.0189489.s017.docx]
