# Supplementary material for: High proportion of genetic cases in patients with advanced cardiomyopathy including a novel homozygous Plakophilin 2-gene mutation
Source: PLoS One. 2017 Dec 18;12(12):e0189489. doi: 10.1371/journal.pone.0189489 (PMC5734774; doi:10.1371/journal.pone.0189489)

**S9 Figure**. **Immunofluorescence staining of myocardial sections using confocal microscopy.** PKP2, connexin43 (CX43), and DAPI staining of left ventricular myocardium of a non-failing (NF, control) and the PKP2 p.His679Tyr mutant heart (family DCM-23, patient III/10, homozygous for PKP2 p.His679Tyr). The control myocardium showed normal myocardial tissue structure whereas selected regions of the failing heart from the PKP2 p.His679Tyr carrier showed significant disarray and misalignment of cardiomyocytes. Of note, immunofluorescence staining of myocardial sections of the PKP2 p.His679Tyr mutant heart showed normal localization of plakophilin and connexin 43 at the intercalated disc when compared to myocardial sections of a rejected donor heart. No fluorescence staining was detectable with the secondary antibody alone (data not shown). Scale bar = 100µm, magnification 1x40.


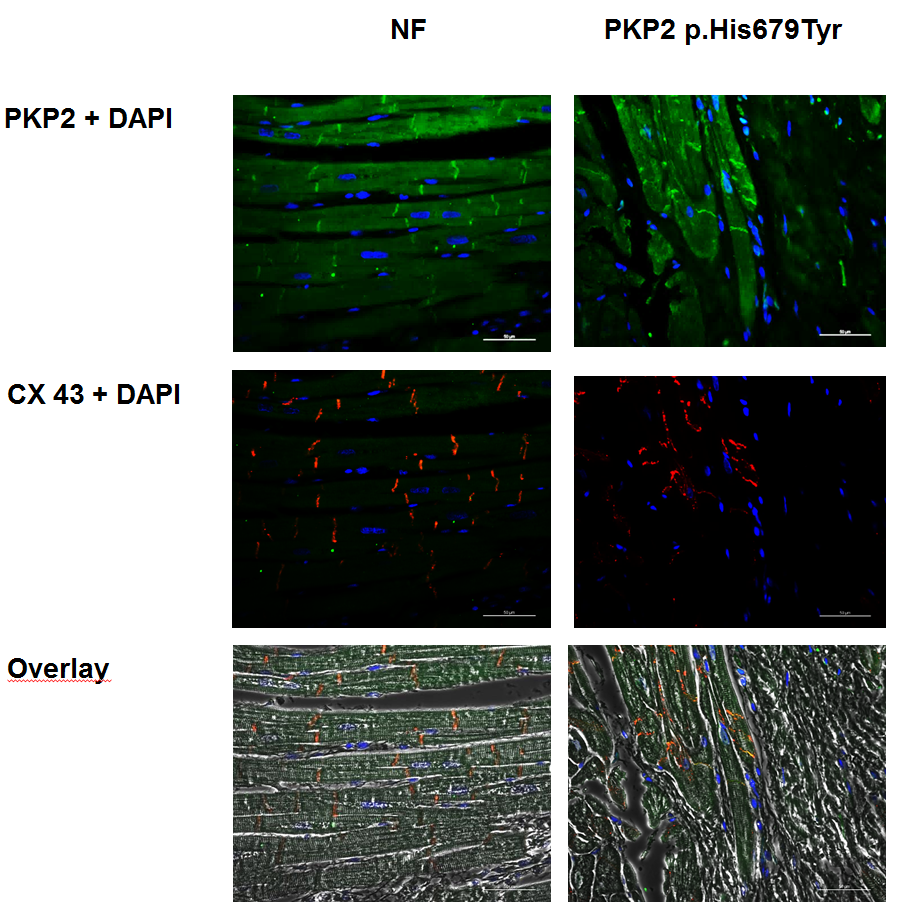

Supplement: S9 Fig — (DOCX) [file pone.0189489.s018.docx]
